# Supramolecular protein assembly in cell-free protein synthesis system

**DOI:** 10.1186/s40643-022-00520-8

**Published:** 2022-03-21

**Authors:** Zhixia Li, Yuting Li, Xiaomei Lin, Yuntao Cui, Ting Wang, Jian Dong, Yuan Lu

**Affiliations:** 1 Tianjin Industrial Microbiology Key Laboratory, College of Biotechnology, Tianjin University of Science and Technology, Tianjin, 300457 China; 2 Key Laboratory of Industrial Biocatalysis, Ministry of Education, Department of Chemical Engineering, Tsinghua University, Beijing, 100084 China

**Keywords:** Cell-free protein synthesis, Protein biomaterials, Supramolecular assembly

## Abstract

Protein-based biomaterials have the characteristics of stability and biocompatibility. Based on these advantages, various bionic materials have been manufactured and used in different fields. However, current protein-based biomaterials generally need to form monomers in cells and be purified before being assembled in vitro. The preparation process takes a long time, and the complex cellular environment is challenging to be optimized for producing the target protein product. Here this study proposed technology for in situ synthesis and assembly of the target protein, namely the cell-free protein synthesis (CFPS), which allowed to shorten the synthesis time and increase the flexibility of adding or removing natural or synthetic components. In this study, successful expression and self-assembly of the dihedral symmetric proteins proved the applicability of the CFPS system for biomaterials production. Furthermore, the fusion of different functional proteins to these six scaffold proteins could form active polymers in the CFPS system. Given the flexibility, CFPS is expected to become a powerful tool as the prototyping and manufacturing technology for protein-based biomaterials in the future.

**Graphical Abstract:**

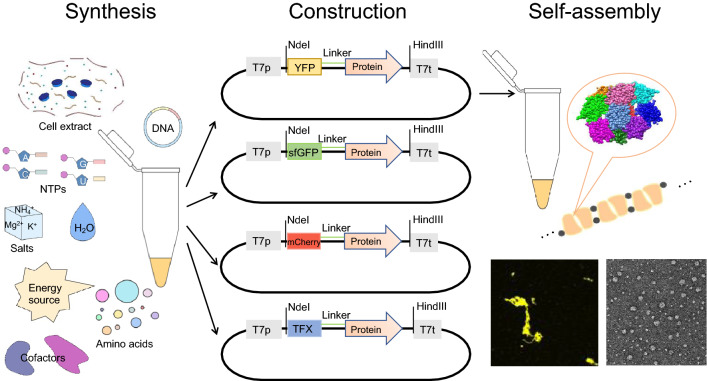

**Supplementary Information:**

The online version contains supplementary material available at 10.1186/s40643-022-00520-8.

## Introduction

Proteins are ubiquitous in nature, and protein self-assembly is the main means to construct the complexity of living systems. Most proteins assemble through non-covalent interactions (hydrophobic interactions, amphiphilic interfaces, hydrogen bond networks, van der Waals interactions, and π–π interactions). With significant structural and functional properties, protein assembly has shown great potential in biocatalysis, material templating, and biomedicine (Wu et al. 2008; Li et al. 2016; Zottig et al. 2020). Protein-based materials have the advantages of biocompatibility, degradability, and good mechanical strength. This has enabled the synthesis of biomaterials using collagen, elastin, and fibrin for multiple biomedical fields such as drug delivery and artificial blood vessel development (Lee et al. 2001; Zhao et al. 2008; Wang et al. 2014; Ozsvar et al. 2015). Some proteins can also be assembled into high-density crystal lattices, which can be used to immobilize enzymes to make them have a longer shelf life or enhance their biocatalytic activity (Tanaka et al. 2010). In addition, proteins can also organize metal, organic and inorganic molecules by assembling different nano-scaffolds as biological templates to create new hybrid materials with complex structures, including nanorings, nanotubes, and nanosheets (Schäffer et al. 2007), which overcomes the heterogeneity and complexity of protein building blocks and increases the material biocompatibility (Ballister et al. 2008; Grigoryan et al. 2011; Kostiainen et al. 2013; Bai et al. 2016; Liu et al. 2016; Oohora et al. 2018). The successful construction of these biological materials provides a new platform for biomedicine and biocatalysis.

The assembly of most homopolymeric proteins found in nature is usually symmetrical. The symmetry of protein allows compact coding of protein components such as viral capsids, cytoskeleton, tubules, and filaments, and symmetric oligomers allow for coordinated, switch-like transformations. Compared with a single protein, this oligomer has a more stable structure and robustness to errors in synthesis, and higher-order symmetry is essential for the construction of large and complex protein assemblies (André et al. 2008; Pagès and Grudinin 2018). The construction of these symmetrical proteins greatly reduces the complexity of the de novo design of self-assembled nanomaterials, provides higher stability and mechanical strength, and enhances the biological functions and physicochemical properties of proteins. At present, more complex protein structures such as polyhedral cages, fibers, loops, tubules, and flat sheets constructed by using protein structural symmetry combined with other non-covalent interactions have been proven (Yeates 2017).

Although biomaterials assembled into symmetrical proteins have made great progress, the complexity of protein synthesis in cells and in vitro self-assembly may limit the rapid and convenient self-assembly of proteins into large polymers and the exploration of the conditions for the formation of macromolecules. The application of cell-free protein synthesis system (CFPS) in the production of complex protein assemblies provides an opportunity to study the assembly process of macromolecules (Dudley et al. 2015; Kelwick et al. 2020; Carlson et al. 2012). First, the CFPS system can quickly synthesize the target protein by adding foreign DNA templates and required nutrients. Second, because it is not restricted by the cell membrane, the synthesis of macromolecular proteins and the self-organizing reaction conditions can be more directly and accurately regulated, so that the assembly process can be studied in a controlled reaction environment (Perez et al. 2016). This technology opens up new ways to manufacture functional biomaterials (Karig et al. 2017; Benítez-Mateos et al. 2018). CFPS has made some progress in the production and assembly of macromolecules (Ramachandran et al. 2008; Lee et al. 2010; Silverman et al. 2020). The five-subunit *E. coli* RNA polymerase and hepatitis B core antigen virus-like particles have been synthesized and assembled (Bundy et al. 2008; Asahara and Chong 2010). The CFPS system has shown great potential in the synthesis and assembly of biomaterials for various applications.

**Fig. 1:**
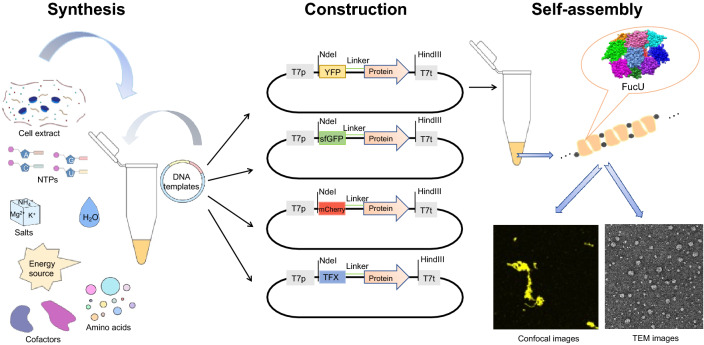
The development of CFPS platform for rapid in situ synthesis and assembly of biomacromolecules

In this study, a novel strategy was proposed, in which DNAs capable of expressing dihedral symmetric polymer proteins were added externally into CFPS systems for in situ expression and assembly (Fig. 1). *E. coli* proteins inducible lysine decarboxylase (LdcI, PDB ID: 3N75), regulatory protein AsnC (AsnC, PDB ID:2CG4), isoaspartyl dipeptidase (IadA, PDB ID: 1POK), l-fucose mutarotase (FucU, PDB ID: 2WCV), 3-methyl-2-oxobutanoate hydroxymethyltransferase (PanB, PDB ID: 1M3U), and biodegradative arginine decarboxylase (AdiA, PDB ID: 2VYC), which have high-order symmetry and can trigger fiber accumulation, were selected in this study (Garcia-Seisdedos et al. 2017). It has been considered that macromolecular polymers need to be fused with different functional proteins to improve the application value of polymers in the future. Self-assembly proteins were first fused with a yellow fluorescent protein (YFP) to prove their expression and self-assembly in the CFPS. Then by changing the redox conditions, the CFPS used to synthesize and express oligomers was optimized, and the salt concentration was adjusted to observe the characteristics of the stability of oligomers. In addition, the fusion protein YFP was also replaced with the green fluorescent protein (sfGFP) and red fluorescent protein (mCherry) to prove the universal applicability of proteins. Finally, the catalytic xylanase (TFX) (Sun et al. 2019), as an important enzyme for converting lignocellulose (a renewable resource), was fused to *E. coli* proteins to verify the functionality.

## Results and discussion

### Determination of fusion protein location

**Fig. 2:**
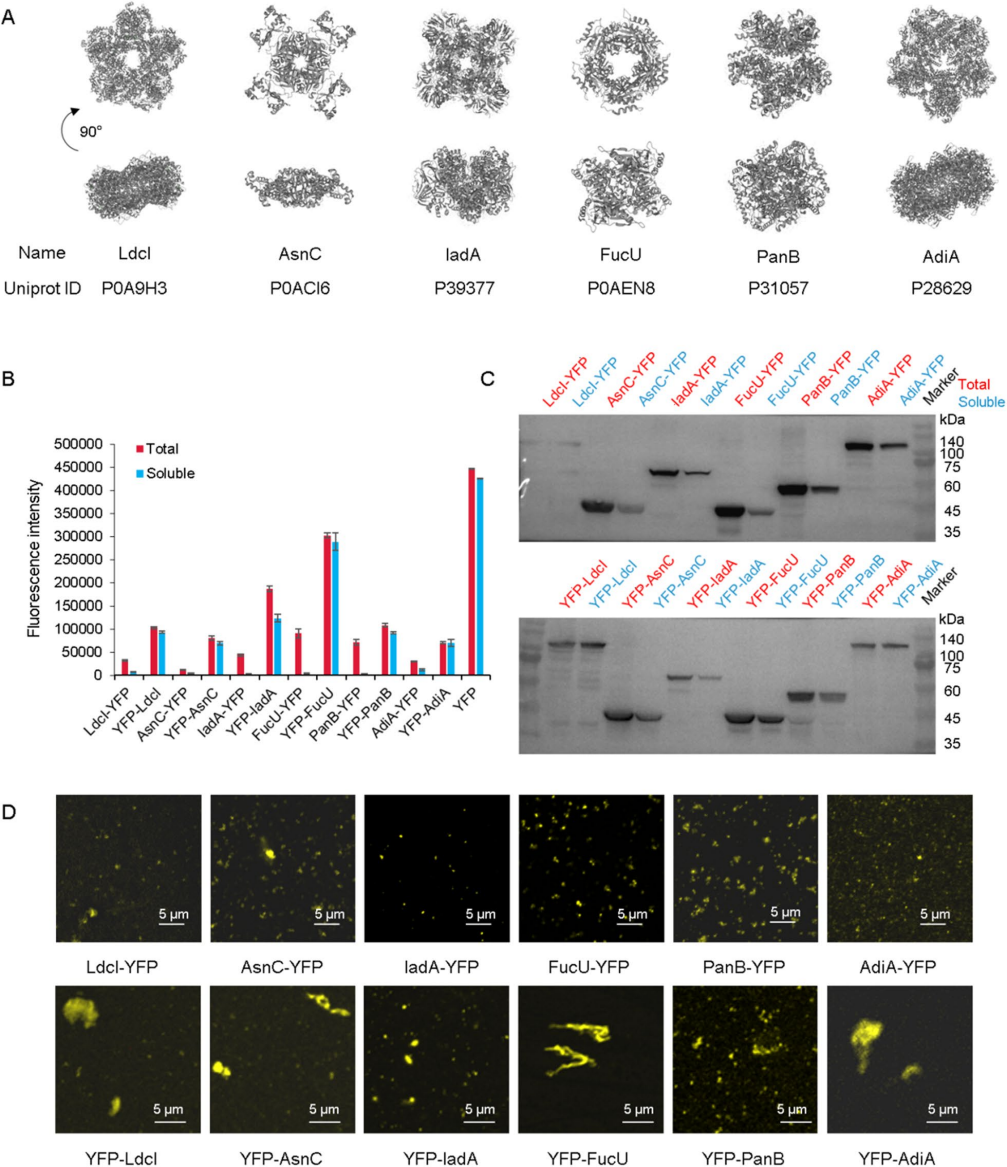
The effect of fusion position of YFP on protein expression and assembly in CFPS system. **A** The structures of six dihedral symmetric proteins. **B** The expression analysis of 12 proteins through the measurement of fluorescence values. **C** The expression analysis of 12 proteins by western blot. **D** The analysis of 12 self-assembled proteins under a confocal microscope

To quickly and conveniently detect the expression and observe the self-assembly form of proteins LdcI, AsnC, IadA, FucU PanB and AdiA in the CFPS system, YFP was first fused to them. However, the influence of the fusion protein YFP on protein self-assembly was unknown. Therefore, YFP was linked to the N-terminus or C-terminus of the proteins, respectively. It could be seen in Fig. 2 that the position of the fusion protein YFP had an effect on the protein expression, and the expression of proteins with YFP at the N-terminus was higher than that at C-terminus. As shown in Fig. 2B and Additional file 1: Table S2, the fluorescence value of proteins with YFP at the N-terminus was 0.5–5 times higher than that at C-terminus. The solubility rate of proteins with YFP at the N-terminus was 1–18 times higher than that at C-terminus. Among different constructs, the protein FucU showed the best expression yield and solubility. Similarly, western blot analysis in Fig. 2C further confirmed that the expression and solubility of proteins with YFP at N-terminus were much better than that at C-terminus, and the protein FucU was great.

Subsequently, the total proteins of these 12 protein constructs were observed through a confocal microscope for analyzing the self-assembly (Fig. 2D, Additional file 1: Fig. S11). The proteins with YFP at N-terminus could self-assemble into 1–10 µm polymers, which were 1–10 times larger than that with YFP at C-terminus. It could also be seen that YFP-FucU could self-assemble into a 10 µm polymer. The other five proteins with different symmetry also showed different assemblies. Overall, by comparing the construct expression, western blot analysis, and confocal microscope observation, the fusion proteins were more suitable for connecting to the N-terminus of scaffold proteins. In addition, the expression and self-assembly of these six proteins fused with N-terminal YFP in the CFPS system were also demonstrated by size-exclusion chromatography. Compared with the YFP protein that did not form a polymer, the other six proteins showed a shorter retention time or a smaller elution volume, which meant that all of them formed high-molecular-weight polymers. (Additional file 1: Fig. S10).

### Analysis of self-assembly protein solubility

**Fig. 3:**
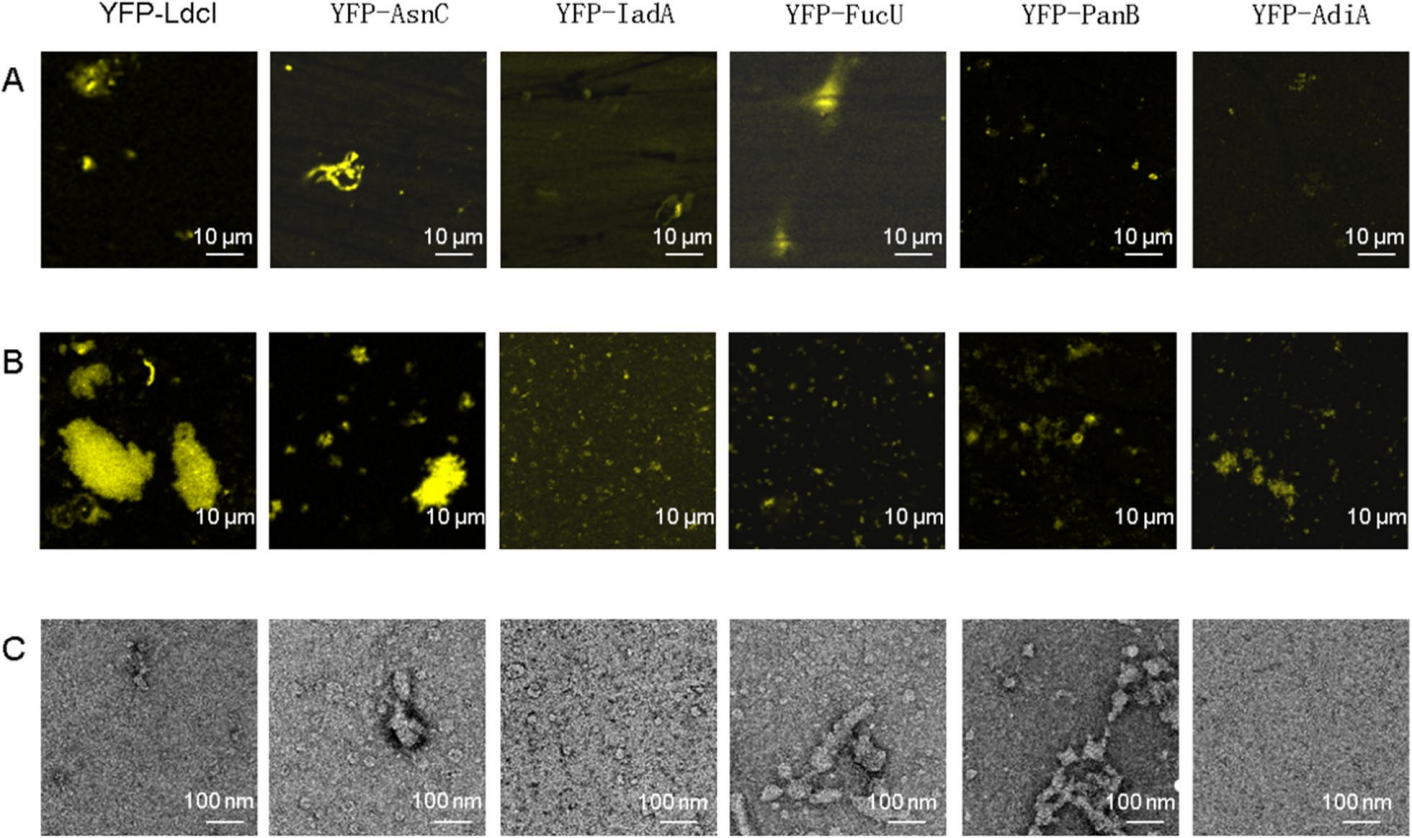
Analysis of six protein constructs. **A** Confocal images of purified soluble proteins of six proteins. **B** Confocal images of the precipitates of six proteins after centrifugation. **C** TEM images of the purified total proteins of six proteins

It has been proved that the solubility of these proteins could reach 60–90%, and the proteins could form large polymers of 10 µm. However, it was not confirmed whether the insoluble precipitate contained large polymers. To understand the self-assembly status of these six proteins more precisely, the protein constructs in the CFPS products were subsequently purified for further analysis. Observed by the confocal microscope, fluorescent macromolecules were seen in both the purified soluble protein and the precipitate after centrifugation (Fig. 3A, B). The biomacromolecules larger than 10 μm were observed in the precipitates of YFP-LdcI and YFP-AsnC. To gain structural insights, these purified proteins were observed under the transmission electron microscope (TEM) (Fig. 3C). However, the polymers were only shown in the purified total proteins and were not observed in the purified precipitates. As shown in Fig. 3C, YFP-AsnC, YFP-FucU, and YFP-PanB could form 100–500 nm protein polymers.

### Effects of CFPS conditions

**Fig. 4:**
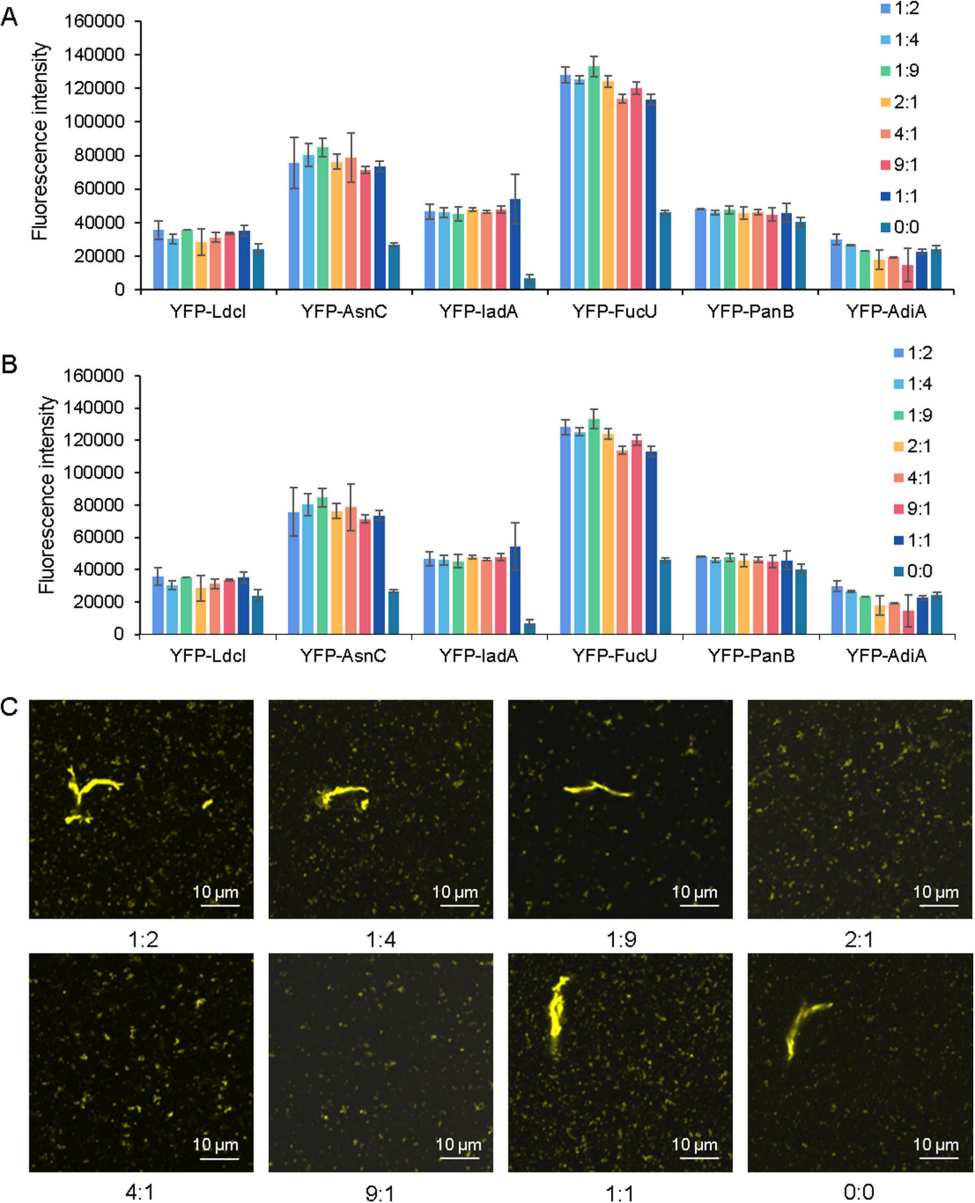
The effects of redox conditions on the self-assembly of protein constructs. **A** The effects of redox conditions on the total protein expression. **B** The effect of redox conditions on the soluble protein expression. **C** The effects of redox conditions on the YFP-FucU, observed from the confocal images

After determining the expression of proteins in the CFPS system, the reaction environment was further explored to improve the expression and assembly. Redox environment affects protein folding by affecting disulfide bond formation (Yin and Swartz 2004; Chakraborty et al. 2021), and therefore a total of 8 different molar ratios of GSSG to GSH (1: 2, 1: 4, 1: 9, 2: 1, 4: 1, 9: 1, 1: 1, and 0: 0) were added into the CFPS system to obtain different redox potentials, and their effects on protein expression and self-assembly were explored. It could be seen that the presence of glutathione increased the expression of YFP-AsnC, YFP-IadA and YFP-FucU (Fig. 4A, B), but had few effects on the expression of other constructs. The presence of glutathione had few effects on the solubility of six proteins. Then YFP-FucU was selected to observe the assembly morphology under the confocal microscope (Fig. 4C). The folded state of YFP-FucU did not change much. It could be seen from the results that the presence of glutathione may affect some elements of the CFPS system, thereby affecting the synthesis and expression of the three proteins YFP-AsnC, YFP-IadA and YFP-FucU. However, changes in these elements had little effect on the expression of the other three proteins and the self-assembly of all proteins. This result also reflected that these six proteins could form more stable states in cell-free systems, thus showing greater potential for forming stable biomaterials.

**Fig. 5:**
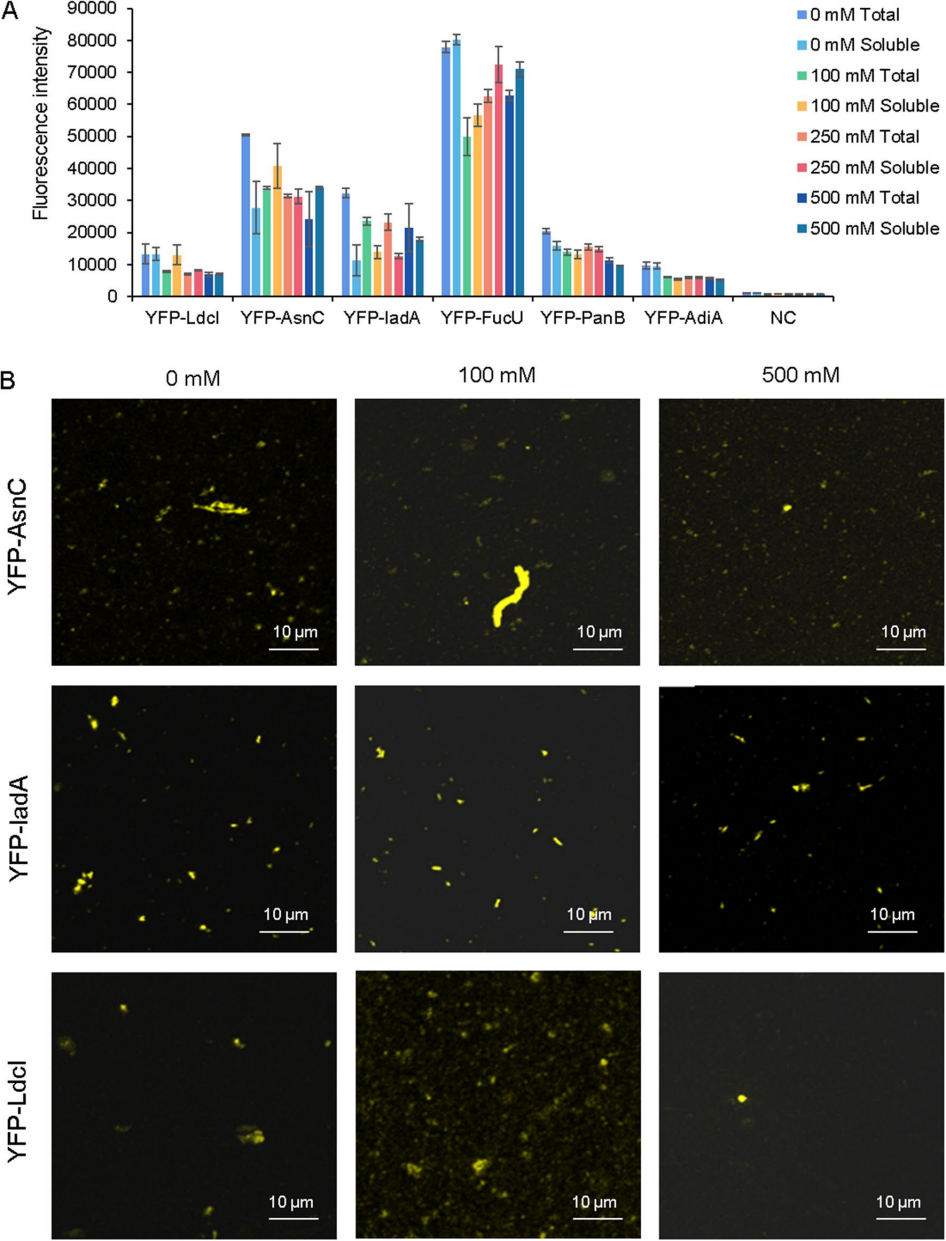
The effects of NaCl concentrations on the expression and self-assembly of proteins. **A** The effects of NaCl concentrations on the expression. **B** The effects of NaCl concentrations on the assembly, observed from the confocal images

It was possible to weaken or enhance the intermolecular electrostatic interactions and hydrophobic interactions by altering the salt concentration of the solution, thereby affecting the protein assembly (Donnarumma et al. 2020; Lefevre et al. 2020; Zhang et al. 2021). So the effect of different concentrations of NaCl (0 mM, 100 mM, 250 mM, and 500 mM) on cell-free protein self-assembly was explored in this article. It could be seen from Fig. 5A that higher salt concentrations could decrease the expression of protein constructs and had few effects on their solubilities. As shown in Fig. 5B, the high concentration of NaCl (500 mM) might disrupt the assembly of proteins. This result showed that these proteins could still form polymers at lower salt concentration, showing a relatively stable state, but the self-assemble effect was weakened at 500 mM salt concentration, which may be due to the fact that the higher salt concentration disrupts the intramolecular interactions of the protein.

### Universal applicability of self-assembly proteins

**Fig. 6:**
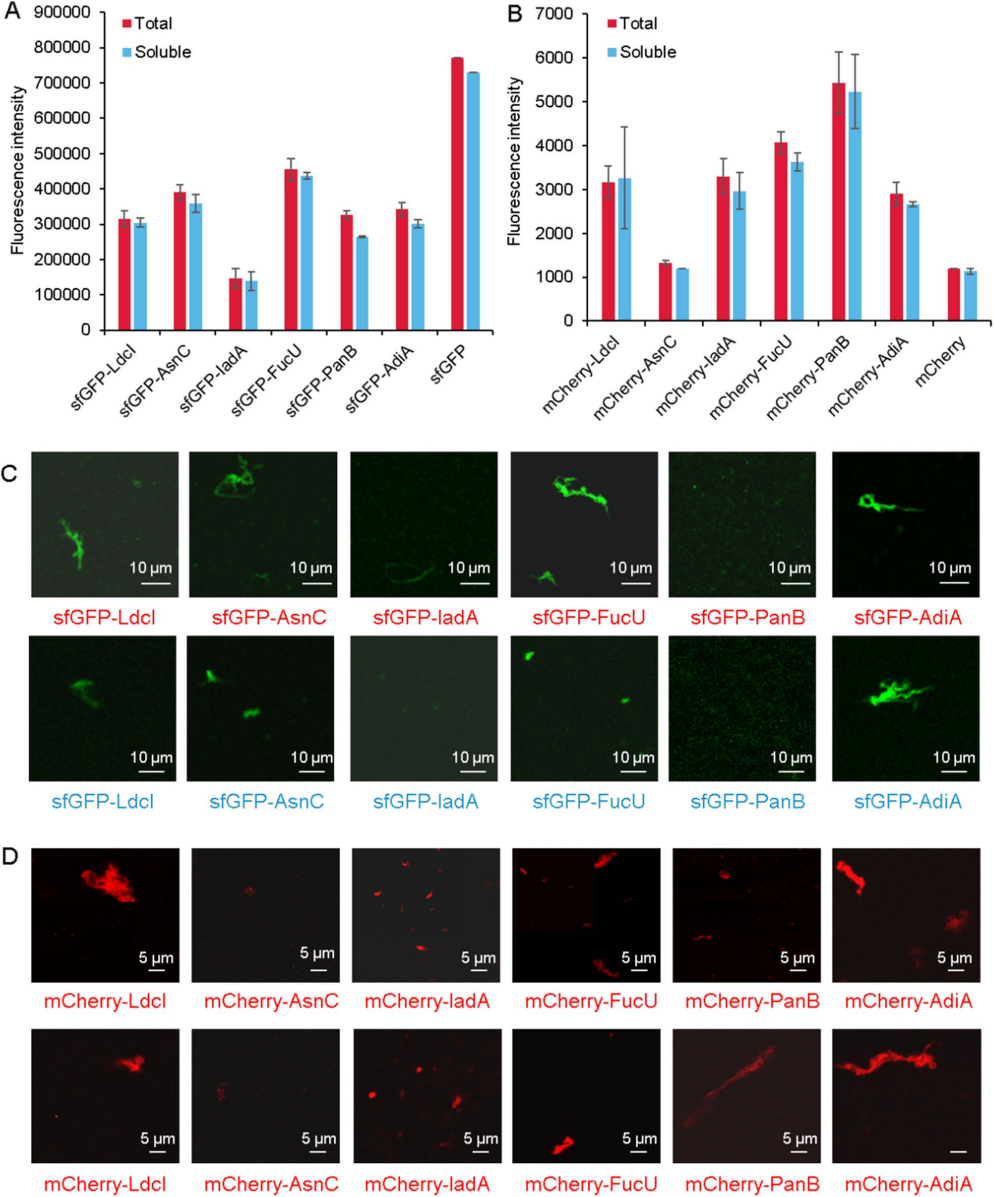
The effects of different fusion proteins on the assembly. **A** Expression of six proteins with sfGFP. **B** Expression of six proteins with mCherry. **C** Confocal imaging of six proteins with sfGFP. The first line referred to the assembly of the total proteins, and the second line referred to the assembly of the soluble proteins. **D** Confocal imaging of six proteins with mCherry. The first line referred to the assembly of the total proteins, and the second line referred to the assembly of the soluble proteins

To explore the effects of replacing the fusion proteins on the self-assembly of proteins, YFP was replaced by sfGFP or mCherry. By measuring the expression fluorescence value (Fig. 6A, B) and observing the self-assembly morphology under the confocal microscope (Fig. 6C, D, Additional file 1: Fig. S11), the universal applicability of these proteins was verified. With the confocal images of proteins with YFP (Additional file 1: Figs. S12–S17), AsnC, FucU, LdcI, and AdiA self-assembled into large polymers of about 10 µm, and IadA and PanB formed small polymers of less than 5 µm. Some proteins were slightly different under confocal microscopy imaging. The reason for this difference is related to the noise generated by the cell-free system and the instrument. From the whole field of view, the fusion of sfGFP and mCherry had no effect on the assembly of the six proteins. All of these results proved that the scaffold proteins in this study could be fused with different functional molecules to form polymers in the CFPS systems for various applications.

### Functional verification of self-assembly proteins

Although the self-assembly properties of these proteins have been characterized, the functionality of the protein polymer needs to be confirmed when the enzymes were fused to the polymer scaffold. To demonstrate their performance, xylanase (TFX) was fused to the N-terminus of the proteins to test its catalytic activity. The TFX is an important enzyme that converts lignocellulose (a high-potential renewable resource) into xylose, and xylose has received widespread attention as an alternative carbon source to replace glucose or starch for different applications, such as biofuels (Sun et al. 2019).

**Fig. 7:**
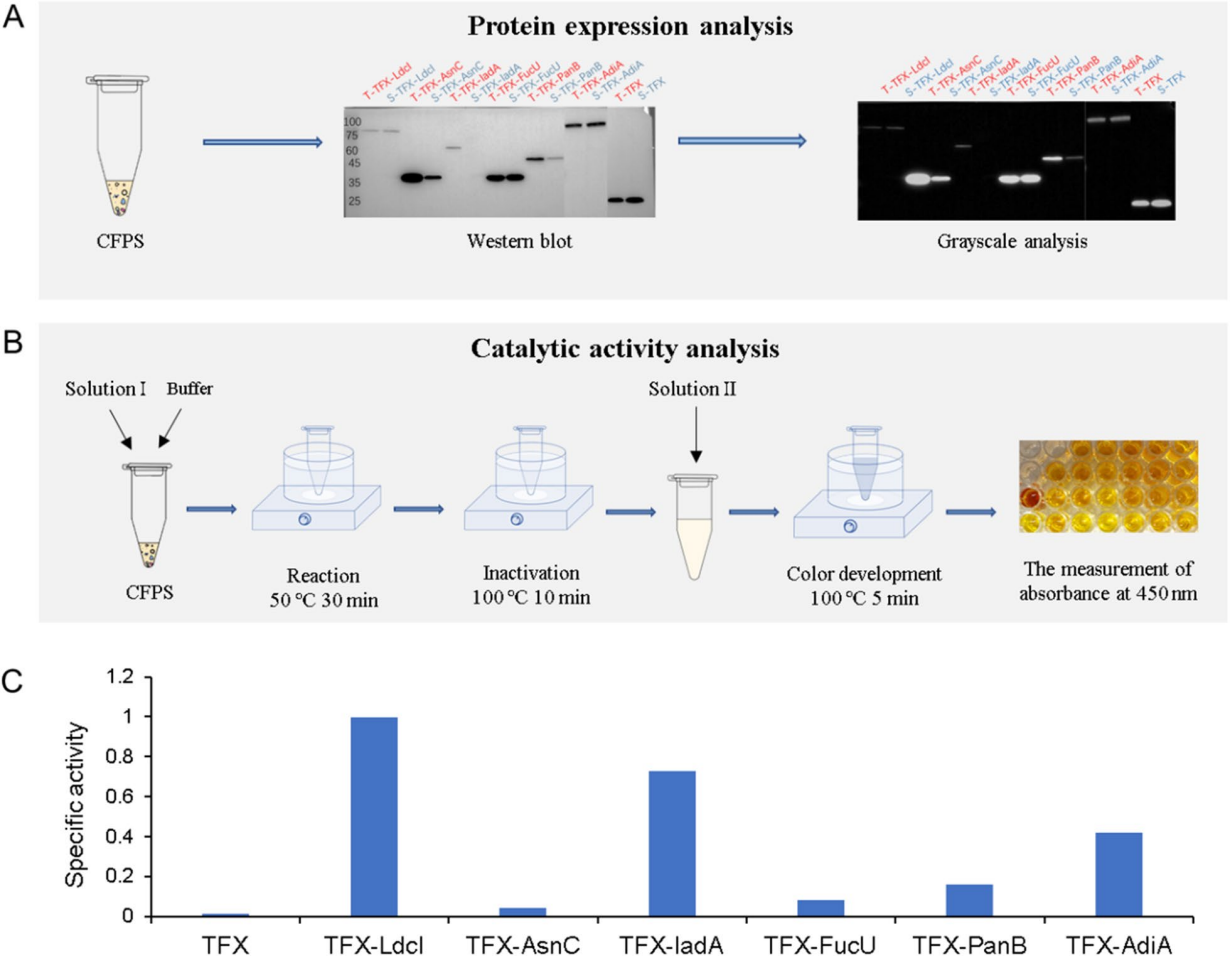
Functional verification of protein polymers fused with enzymes. **A** Schematic diagram of protein expression analysis. **B** Schematic diagram of enzyme catalytic activity analysis. **C** Specific activity analysis of six TFX–protein constructs

The proteins carrying TFX were expressed and self-assembled in the CFPS system, and the protein expression analysis (Fig. 7A, Additional file 1: Fig. S18) and catalytic activity analysis (Fig. 7B) were performed. The specific enzyme activities of 6 constructs were analyzed, as shown in Fig. 7C. The specific activities of TFX enzymes fused with different proteins were different, which might be attributed to different structural effects, but they were all higher than that of TFX alone. This may be due to the steric proximity effect caused by the ordered linkage with symmetric protein scaffold, the specific activity of the enzyme was related to its spatial structure, such that TFX fused to the six proteins more active than TFX alone (Huang et al. 2012). These results validated that the supramolecular assembly of proteins could be used as polymer scaffolds for biocatalysis applications.

### Comparison of six proteins expressing and self-assembling in cell-free or cell systems

**Fig. 8:**
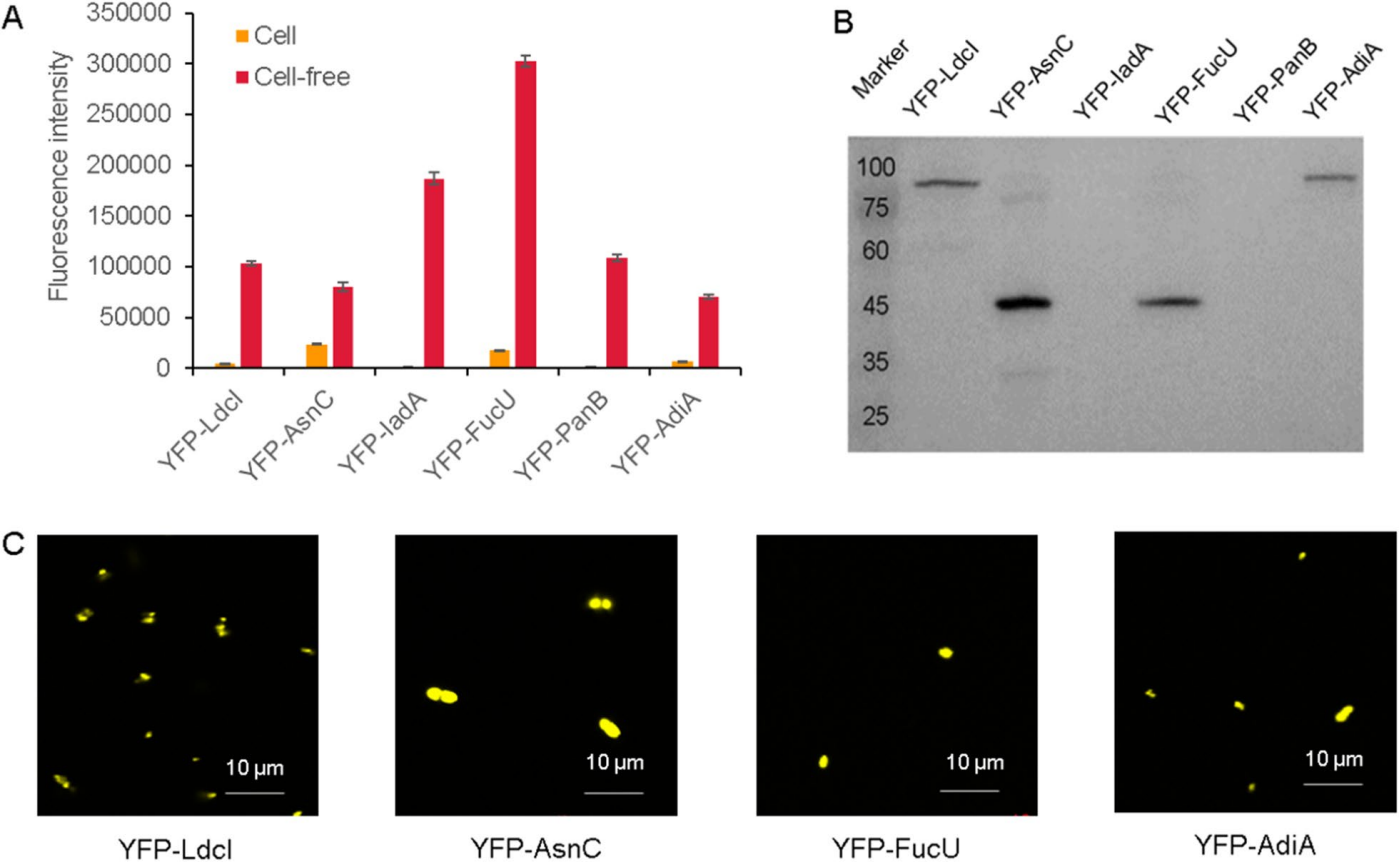
Expression and self-assembly of six proteins in cells. **A** Comparison of the fluorescence level of proteins in cell culture and the CFPS systems. **B** The expression analysis of six proteins expressed in cells by western blot. **C** Imaging of protein self-assembly in cells under confocal microscopy

All six proteins selected in this work were also expressed and self-assembled in *E. coli* cells. However, these cells grew slowly, which demonstrated that these proteins might have toxic or inhibitory effects on the cell growth. According to the fluorescence level, it could be seen that the expression of these proteins in cells were low, as the highest fluorescence in cell culture was only 26,500 (YFP-AdiA), which was much lower than the fluorescence level of the proteins expressed in the CFPS system (Fig. 8A). Combined with the results of the western blot analysis, it was found that YFP-IadA and YFP-PanB were not expressed well (Fig. 8B). The intracellular self-assembly of these proteins was also observed under confocal fluorescence microscopy. Due to the tethering of the cell membrane, these proteins were only able to form aggregates no larger than 5 µm (Fig. 8C).Therefore, it was obvious that the CFPS system was more suitable for the controllable expression and forming larger polymers.

## Conclusion

In this study, six *E. coli* proteins that had symmetry were successfully expressed and assembled into polymers larger than 1 µm in the CFPS system. Among them, the protein FucU with YFP fused to the N-terminus showed great expression and solubility, and could be assembled into large polymer of about 10 μm. These polymers could serve as a biological template to organize inorganic or organic molecules, thereby creating novel hybrid materials with complex structures and high biocompatibility. This would provide new platforms for biomedicine and biocatalysis. The catalytically active enzyme TFX was also fused to proteins to prove their functions of proteins as scaffolds. These results fully illustrated the flexible CFPS system for in situ expression and assembly of proteins, and provided a high-quality platform for the faster and more convenient production of biomaterials. Although expressing in the CFPS system directly is more expensive than expressing the protein intracellularly and then assembling it in vitro, its advantages lie in the ability to regulate and express the protein in situ and self-assemble in CFPS. This technology does not require the large-scale expression of purified protein and is more accurate, which is very suitable for the basic study or prototyping design. Cost is one of the main concerns for biomanufacturing, and to realize it, much more work needs to be done to reduce the making cost of CFPS systems. There is no doubt that cell-free systems have great potential in investigating the supramolecular assembly of proteins and producing functional biomaterials.

## Methods and materials

### Strains and plasmid construction

The *E. coli* strains were used in this study, including BL21 (DE3), DH5α, and Rosetta (DE3). DH5α was used for plasmid cloning, BL21 (DE3) was used for the preparation of T7 RNA polymerase, and Rosetta (DE3) was used for preparing cell extracts. The genes of proteins carrying YFP or xylanase (TFX) were synthesized by Azenta company, and pET23a plasmids were used as the backbone. The plasmids of proteins fused with sfGFP or mCherry were constructed by cloning technology, and the gene sequences were confirmed by TianYi Biotechnology. The specific gene fragment sequences and amino acid sequences are shown in Supplementary material Additional file 1: Tables S1, S2, and the protein structure design diagram and six plasmid maps are shown in Additional file 1: Figs. S1–S6.

### Cell extract preparation

For standard cell-free extract preparation, *E. coli* Rosetta (DE3) cells were cultivated in 4 L 2xYTP medium (1.6% tryptone; 1% yeast extract; 0.5% sodium chloride; 40 mM K_2_HPO_4_; 22 mM KH_2_PO_4_) at 37 °C, 220 rpm. Cells were then collected in the late logarithmic growth phase (∼4 h, OD600 = 2). The cell pellet was washed 3 times with pre-cooled S30A buffer (14 mM magnesium glutamate; 60 mM potassium glutamate; 50 mM Tris, pH 7.7). The cells were resuspended in S30A buffer (1 mL buffer per 1 g wet cells) and disrupted in a high-pressure homogenizer (1000 bar). After the lysate was centrifuged at 12,000×*g* for 10 min at 4 °C, the supernatant was aspirated, and 3 mM DTT was added. Then the samples were incubated at 37 °C for 80 min and centrifuged at 12,000×*g* at 4 °C for 10 min. Then the supernatant was transferred to the dialysis membrane tube (6 ~ 8 kDa relative molecular mass), which was dialyzed for 3 h at 4 °C. The dialysate was then centrifuged at 12,000×*g* for 10 min at 4 °C, and the supernatant was extracted, flash frozen, and stored at − 80 °C (Lin et al. 2020).

### Cell-free reaction

Generally, the cell-free reaction mixture included the following components: 30% S30 cell extract (v/v%); 15–20 ng/µL DNA templates; 175 mM potassium glutamate; 10 mM ammonium glutamate; 2.7 mM potassium oxalate monohydrate; 10 mM magnesium glutamate; 50 mM each of 19 amino acids without glutamic acid; 3 mM phosphoenol pyruvate (PEP); 1 mM putrescine; 1.5 mM spermidine; 0.33 mM nicotinamide adenine dinucleotide (NAD); 1.2 mM ATP; 0.86 mM each of CTP, GTP and UTP; 0.27 mM coenzyme A; 170 µg/mL tRNA; 34 µg/mL folinic acid; T7 RNA polymerase prepared from *E. coli* BL21 (DE3) cell extract. The reaction system was assembled on ice. After the assembly was completed, the insoluble matter was removed by centrifugation, and then the mixture was aliquoted into 1.5 mL EP tubes. Finally, plasmid DNA was added to the reaction system and incubated at 30 °C for 12 h. The specific CFPS workflows are shown in the Additional file 1: Figs. S7–S9.

### Protein purification

Since the protein self-assembled to form a polymer, the magnetic purification method was used to purify the protein in this study. The magnetic bead solution was placed on a vortex mixer and mixed well. A pipette was used to draw 200 µL of the magnetic bead suspension into a 1.5 mL EP tube for magnetic separation and discarded the supernatant. Then 200 µL of binding buffer (20 mM phosphate buffer, 500 mM NaCl, 50 mM imidazole, pH 7.4) was added to the tube, turned the centrifuge tube up and down to resuspend the magnetic beads, then performed magnetic separation, removed the supernatant, and repeated the washing twice. Then the sample was added to a centrifuge tube containing magnetic beads, shook with a vortex mixer for 15 s, placed the centrifuge tube on a rotary mixer, and rotated at room temperature for 30 min. Then magnetic separation was performed, the supernatant discarded, and 400 µL washing buffer (20 mM phosphate buffer, 500 mM NaCl, 100 mM imidazole, pH 7.4) added into a centrifuge tube. Gently flipped to resuspend the magnetic beads, performed magnetic separation, and repeated the washing twice. Then 400 µL washing buffer was added and the magnetic bead suspension was transferred to a new centrifuge tube. Magnetic separation was performed again, and the supernatant was discarded. Then 40 µL elution buffer (20 mM phosphate buffer, 500 mM NaCl, 500 mM imidazole, pH 7.4) was added to elute the protein bound to the magnetic beads to obtain a purified target protein sample. The purified protein was dialyzed against 20 mM Tris buffer (pH 7.5) and stored at − 80 °C.

### Protein characterization

*Fluorescence measurement.* The CFPS products were diluted 100 times and measured on the plate reader (TECAN FINFORM M200Pro). The excitation wavelengths of green, red, and yellow fluorescences were 485 nm, 587 nm, and 500 nm, respectively, and the corresponding emission wavelengths were 520 nm, 615 nm, and 530 nm, respectively. The experiments were carried out at least three times.

*Fluorescence microscopy imaging*. After the CFPS was completed, 10 μL of the reaction mixture was placed on a Petri dish. Bright-field transmission and fluorescence images were obtained through a confocal microscope (Zeiss LSM880) (Zhang et al. 2020). The samples were diluted 10 times with ddH_2_O.

*TEM observation*. 7 μL of protein sample was drawn from the protein stored in 20 mM Tris, pH 7.5, and placed on the copper mesh of transmission electron microscope that had been hydrophilized for 30 s. The excess liquid was absorbed, dried for 1 min, and uranyl acetate solution used for 30 s. The excess liquid was sucked off and dried for 1 min. The sample was observed in a projection cryo-electron microscope.

### Western blot analysis

The sixfold diluted sample was denatured at 98 °C for 10 min. Then it was loaded into SDS-PAGE gel and separated at 120 V, 200 mA. After the electrophoresis separation, the protein was transferred from the gel to the PVDF membrane through a current of 120 V and 300 mA. The membrane was sealed in the TBST buffer with milk for 1 h. Then the membrane was incubated in anti-His tag mouse polyclonal antibody solution at 4 °C overnight and washed with the TBST buffer 3 times after incubation. There was a 10-min incubation between each wash. Then the membrane was incubated in anti-mouse IgG antibody solution for 40 min, washed three times with the TBST buffer, and incubated for 10 min between each wash. MiniChem instrument was used for imaging.

### Measurement of enzyme activity

Neutral xylanase (NEX) catalyzed the degradation of xylan into reducing oligosaccharides and monosaccharides in a neutral environment, and further reacted with 3,5-dinitrosalicylic acid under boiling water bath conditions. There was a characteristic absorption peak at 540 nm, and the color of the reaction solution was proportional to the amount of reducing sugar produced by enzymatic hydrolysis. The enzyme activity could be calculated by measuring the increase rate of the absorbance of the reaction solution at 540 nm. Three parallel experiments were done for each enzyme.

### Supplementary Information

**Additional file 1: Table S1.** The genetic sequences used in this study. **Table S2.** The amino acid sequences used in this study. **Table S3.** Fluorescence ratios of six proteins fused with YFP at the N-terminus to YFP at the C-terminus. **Table S4.** Structural information of six proteins. **Fig. S1.** The protein structure design diagram. **Fig. S2.** The plasmid map of pET23a-mCherry-FucU. **Fig. S3.** The plasmid map of pET23a-sfGFP-FucU. **Fig. S4.** The plasmid map of pET23a-TFX-FucU. **Fig. S5.** The plasmid map of pET23a-YFP-FucU. **Fig. S6.** The plasmid map of pET23a-FucU-YFP. **Fig. S7.** The workflow of cell-free protein synthesis system. **Fig. S8.** The workflow for the effects of redox environments on cell-free protein expression and self-assembly. **Fig. S9.** The workflow for the effects of NaCl concentration on cell-free protein expression and self-assembly. **Fig. S10.** The size-exclusion chromatography (SEC) results of six proteins. **Fig. S11.** Imaging of YFP, sfGFP, mCherry proteins expressed individually in CFPS system under confocal microscope. **Fig. S12.** Imaging of the self-assembled protein YFP-LdcI under a confocal microscope. **Fig. S13.** Imaging of the self-assembled protein YFP-AsnC under a confocal microscope. **Fig. S14.** Imaging of the self-assembled protein YFP-IadA under a confocal microscope. **Fig. S15.** Imaging of the self-assembled protein YFP-FucU under a confocal microscope. **Fig. S16.** Imaging of the self-assembled protein YFP-PanB under a confocal microscope. **Fig. S17.** Imaging of the self-assembled protein YFP-AdiA under a confocal microscope. **Fig. S18.** Western blot analysis of proteins fused with TFX.

## Data Availability

All data and materials are available in the manuscript and supporting information.

## Abbreviations

CFPS: Cell-free protein synthesis
YFP: Yellow fluorescent protein
sfGFP: Superfolder green fluorescent protein
mCherry: Red fluorescent protein
PEP: Phosphoenolpyruvate
NEX: Neutral xylanase
TFX: *Thermobifida fusca* Xylanase
