# Supplementary material for: Supramolecular protein assembly in cell-free protein synthesis system
Source: Bioresour Bioprocess. 2022 Mar 21;9(1):28. doi: 10.1186/s40643-022-00520-8 (PMC10991650; doi:10.1186/s40643-022-00520-8)
Supplement: Supplementary file 1 — Additional file 1: Table S1. The genetic sequences used in this study. Table S2. The amino acid sequences used in this study. Table S3. Fluorescence ratios of six proteins fused with YFP at the N-terminus to YFP at the C-terminus. Table S4. Structural information of six proteins. Fig. S1. The protein structure design diagram. Fig. S2. The plasmid map of pET23a-mCherry-FucU. Fig. S3. The plasmid map of pET23a-sfGFP-FucU. Fig. S4. The plasmid map of pET23a-TFX-FucU. Fig. S5. The plasmid map of pET23a-YFP-FucU. Fig. S6. The plasmid map of pET23a-FucU-YFP. Fig. S7. The workflow of cell-free protein synthesis system. Fig. S8. The workflow for the effects of redox environments on cell-free protein expression and self-assembly. Fig. S9. The workflow for the effects of NaCl concentration on cell-free protein expression and self-assembly. Fig. S10. The size-exclusion chromatography (SEC) results of six proteins. Fig. S11. Imaging of YFP, sfGFP, mCherry proteins expressed individually in CFPS system under confocal microscope. Fig. S12. Imaging of the self-assembled protein YFP-LdcI under a confocal microscope. Fig. S13. Imaging of the self-assembled protein YFP-AsnC under a confocal microscope. Fig. S14. Imaging of the self-assembled protein YFP-IadA under a confocal microscope. Fig. S15. Imaging of the self-assembled protein YFP-FucU under a confocal microscope. Fig. S16. Imaging of the self-assembled protein YFP-PanB under a confocal microscope. Fig. S17. Imaging of the self-assembled protein YFP-AdiA under a confocal microscope. Fig. S18. Western blot analysis of proteins fused with TFX. [file 40643_2022_520_MOESM1_ESM.docx]

**Supporting Information**

**Supramolecular protein assembly in cell-free protein synthesis system**

**Zhixia Li^1,2,#^, Yuting Li^1,2,#^, Xiaomei Lin^2,#^, Yuntao Cui^2^, Ting Wang^2^, Jian Dong^1,^*, Yuan Lu^2,^***

1 Tianjin Industrial Microbiology Key Laboratory, College of Biotechnology, Tianjin University of Science and Technology, Tianjin 300457, China

2 Key Laboratory of Industrial Biocatalysis, Ministry of Education, Department of Chemical Engineering, Tsinghua University, Beijing 100084, China

# These authors contributed equally.

*Corresponding authors. Email: yuanlu@tsinghua.edu.cn (Y. Lu),

dongjian@tust.edu.cn (J. Dong)

**Table S1** The genetic sequences used in this study.

| Name | Sequence |
| --- | --- |
| LdcI | ATGAACGTGATTGCGATTCTGAACCACATGGGCGTGTATTTTAAAGAAGAACCGATTCGCGAACTGCATCGCGCGCTGGAACGCCTGAACTTTCAGATTGTGTATCCGAACGATCGCGATGATCTGCTGAAACTGATTGAAAACAACGCGCGCCTGTGCGGCGTGATTTTTGATTGGGATAAATATAACCTGGAACTGTGCGAAGAAATTAGCAAAATGAACGAAAACCTGCCGCTGTATGCGTTTGCGAACACCTATAGCACCCTCGATGTCAGCCTTAACGATCTGCGCCTGCAGATTAGCTTTTTTGAATATGCGCTGGGCGCGGCGGAAGATATTGCGAATAAAATAAAGCAGACCACCGATGAATATATTAACACCATTCTGCCGCCGCTGACCAAAGCACTGTTCAAGTATGTGCGCGAAGGCAAATATACCTTTTGCACCCCGGGCCACATGGGCGGCACCGCGTTTCAGAAATCCCCCGTGGGCAGCCTGTTTTATGACTTTTTTGGCCCGAACACCATGAAAAGCGATATTAGCATTAGCGTGAGCGAACTGGGCAGCCTGCTGGATCATAGCGGCCCGCATAAAGAAGCGGAACAGTATATTGCGCGCGTGTTTAACGCGGATCGCAGCTATATGGTGACCAACGGCACGAGCACCGCGAACAAAATAGTAGGCATGTATAGCGCGCCGGCGGGCAGCACCATTCTGATTGATCGCAACTGCCATAAAAGCCTGACCCATCTGATGATGATGAGCGATGTGACCCCGATTTATTTTCGCCCGACCCGCAACGCGTATGGCATTCTGGGCGGCATTCCGCAGAGCGAATTTCAGCATGCGACCATTGCGAAACGCGTGAAAGAAACCCCGAACGCGACCTGGCCGGTGCATGCGGTGATTACCAACAGCACCTATGATGGCCTGCTGTATAACACCGATTTTATTAAGAAAACCCTGGATGTGAAGAGTATTCATTTTGATAGCGCGTGGGTGCCGTATACCAACTTTAGCCCGATTTATGAAGGCAAATGCGGCATGAGCGGCGGCCGCGTGGAAGGCAAAGTGATTTATGAAACGCAGAGCACCCATAAACTGCTGGCGGCGTTTAGCCAAGCGAGCATGATACATGTGAAGGGCGATGTGAACGAAGAAACCTTTAACGAAGCGTATATGATGCATACCACCACGAGCCCGCATTACGGAATTGTGGCGAGTACCGAAACCGCGGCGGCGATGATGAAAGGCAACGCGGGCAAACGCCTGATTAACGGCAGCATTGAACGCGCGATTAAATTTCGCAAAGAAATTAAACGCCTGCGCACCGAAAGCGATGGCTGGTTTTTTGATGTGTGGCAGCCGGATCATATTCTGACCACCGAATGCTGGCCGCTGCGCAGCGATAGCACCTGGCATGGCTTTAAAAACATTGATAACGAACACATGTATCTGGATCCGATTAAAGTGACCCTGCTGACCCCGGGCATGGAAAAAGATGGCACCATGAGCGATTTTGGCATTCCGGCGAGCATTGTGGCGAAATATTTAGATGAACATGGAATTGTGGTGGAAAAAACCGGCCCGTATAACCTGCTGTTTCTGTTTAGCATTGGCATTGATAAAACCAAAGCGCTGAGCCTGCTGCGCGCGCTGACCGATTTTAAACGCGCGTTTGATCTGAACCTGCGCGTGAAAAACATGCTGCCGAGCCTGTATCGCGAAGATCCGGAATTTTATGAAAACATGCGCATTCAAGAACTGGCGCAGAACATTCATAAACTGATTGTGCATCATAACCTGCCGGATCTGATGTATCGCGCGTTTGAAGTGCTGCCGACCATGGTGATGACCCCGTATGCGGCGTTTCAGAAGGAGCTGCATGGCATGACCGAAGAAGTGTACTTAGATGAAATGGTGGGCCGCATTAACGCGAACATGATTCTGCCGTATCCGCCGGGCGTGCCGCTGGTGATGCCGGGCGAAATGATTACCGAAGAAAGCCGCCCGGTGCTGGAATTTCTGCAGATGCTGTGCGAAATTGGCGCGCATTATCCGGGCTTTGAAACCGATATTCATGGCGCGTATCGCCAAGCGGATGGCCGCTATACCGTGAAAGTGCTGAAAGAAGAAAGCAAAAAG |
| AsnC | ATGGAAAACTATCTGATTGATAACCTGGATCGCGGCATTCTGGAAGCGCTGATGGGCAACGCGCGCACCGCGTATGCGGAACTGGCGAAACAGTTTGGCGTGAGCCCGGAAACCATTCATGTGCGCGTGGAAAAAATGAAACAAGCGGGCATTATTACCGGCGCGCGCATTGATGTGAGCCCGAAACAGCTGGGCTATGATGTGGGCTGCTTTATTGGCATTATTCTGAAAAGCGCGAAAGATTATCCGAGCGCGCTGGCGAAACTGGAAAGCCTGGATGAAGTGACCGAAGCGTACTATACCACCGGCCACTATAGCATTTTTATTAAAGTGATGTGCCGCAGCATTGATGCGCTGCAGCATGTGCTGATTAACTATATTCAGACCATTTATGAAATTCAGAGCACCGAAACCCTGATTGTGCTGCAGAACCCGATTATGCGCACCATTAAACCG |
| IadA | ATGATTGACTATACCGCGGCGGGCTTTACCCTGCTGCAAGGCGCGCATCTGTATGCGCCGGAAGATCGCGGCATTTGCGATGTGCTGGTGGCGAACGGCAAAATTATTGCGGTGGCGAGCAACATTCCGAGCGATATTGTGCCGAACTGCACCGTGGTGGATCTGAGCGGTCAGATTCTGTGCCCGGGCTTTATTGATCAACATGTGCATCTGATTGGCGGTGGAGGAGAGGCCGGCCCAACCACCCGCACCCCCGAAGTGGCTCTGAGCCGCCTGACCGAAGCGGGCGTGACGAGCGTGGTGGGCTTACTTGGCACAGACAGCATTAGCCGCCATCCGGAAAGCCTGCTGGCGAAAACCCGCGCGCTGAACGAAGAAGGCATTAGCGCGTGGATGCTGACCGGCGCGTATCATGTGCCGAGCCGCACCATTACCGGCAGCGTGGAAAAAGATGTGGCGATTATTGATCGCGTGATTGGCGTGAAATGCGCGATTAGCGATCATCGCAGCGCGGCGCCGGATGTGTATCATCTGGCGAACATGGCGGCGGAAAGCCGCGTGGGCGGCCTGCTGGGTGGTAAACCGGGCGTGACCGTGTTTCACATGGGCGATAGCAAAAAAGCGCTGCAGCCGATTTATGATCTGCTGGAAAACTGCGATGTGCCGATTAGCAAACTGCTGCCGACCCATGTGAACCGCAACGTGCCGCTGTTTTATCAAGCGCTGGAATTTGCGCGCAAAGGCGGCACCATTGATATTACGAGCAGCATTGATGAACCTGTGGCTCCTGCAGAAGGCATCGCGCGCGCTGTGCAAGCGGGCATTCCGCTGGCGCGCGTGACCCTGAGCAGCGATGGCAACGGCAGTCAGCCGTTTTTTGATGATGAAGGCAACCTGACCCATATTGGGGTCGCGGGCTTTGAAACCCTGCTGGAAACCGTGCAAGTGCTGGTGAAAGATTATGATTTTAGCATTAGCGATGCGCTGCGCCCGCTGACGAGCTCCGTGGCGGGGTTCCTGAATCTGACCGGCAAAGGCGAAATTCTGCCGGGCAACGATGCGGATCTGCTGGTGATGACCCCGGAACTGCGCATTGAACAAGTGTATGCGCGCGGCAAACTGATGGTGAAAGATGGCAAAGCGTGCGTGAAAGGCACCTTTGAAACCGCG |
| FucU | ATGCTGAAAACCATTAGCCCGCTGATTAGCCCGGAACTGCTGAAAGTGCTGGCGGAAATGGGCCATGGCGATGAAATTATTTTTAGCGATGCGCATTTTCCGGCGCATAGCATGGGCCCGCAAGTGATTCGCGCGGATGGCCTGCTGGTGAGCGATCTGCTGCAAGCGATTATTCCGCTGTTTGAACTGGATAGCTATGCGCCGCCGCTGGTGATGATGGCGGCGGTGTATGGCGATACCCTGGATCCGGAAGTGGAACGCCGCTATCGCAACGCGCTGAGCCTGCAAGCGCCGTGCCCGGATATTATTCGCATTAACCGCTTTGCGTTTTATGAACGCGCGCAGAAAGCGTTTGCGATTGTGATTACCGGCGAACGCGCGAAATATGGCAACATTCTGCTGAAAAAAGGCGTGACCCCG |
| PanB | ATGAAACCGACCACCATTAGCCTGCTGCAGAAATATAAACAAGAGAAAAAACGCTTTGCGACCATTACCGCGTATGACTATAGCTTTGCGAAACTGTTTGCGGATGAAGGCCTGAACGTGATGCTGGTGGGCGATAGCCTGGGCATGACCGTGCAAGGCCATGATAGCACCCTGCCGGTGACCGTGGCGGATATTGCGTATCATACCGCTGCGGTTCGCCGTGGTGCGCCGAACTGCCTGCTGCTGGCGGATCTGCCGTTTATGGCGTATGCGACCCCGGAACAAGCGTTTGAAAACGCGGCGACCGTGATGCGCGCGGGCGCGAACATGGTGAAAATTGAAGGCGGCGAATGGCTGGTGGAAACCGTGCAGATGCTGACCGAACGCGCGGTGCCGGTGTGCGGCCATCTGGGCCTGACCCCGCAGAGCGTGAACATTTTTGGAGGCTACAAGGTGCAAGGCCGCGGTCTTCTGGCGGGCCTTCAGCTATTAAGCGATGCGCTGGCGCTGGAAGCAGCGGGCGCTCAGCTGCTGGTGCTGGAATGCGTGCCGGTGGAACTGGCGAAACGCATTACCGAAGCGCTGGCGATTCCGGTGATTGGCATTGGCGCGGGCAACGTGACCGATGGTCAGATTCTGGTGATGCATGATGCGTTTGGCATTACCGGCGGCCATATTCCGAAATTTGCGAAAAACTTTCTGGCGGAAACCGGCGATATTCGCGCGGCTGTCCGTCAGTACATGGCGGAAGTGGAAAGCGGCGTGTATCCGGGCGAAGAACATAGCTTTCAT |
| AdiA | ATGAAAGTGCTGATTGTGGAAAGCGAATTTCTGCATCAAGATACCTGGGTGGGCAACGCGGTGGAACGCCTGGCGGATGCGCTTAGTCAGCAGAACGTGACCGTGATTAAAAGCACGAGCTTTGATGATGGCTTTGCGATTCTGAGCAGCAACGAAGCGATTGATTGCCTGATGTTTAGCTATCAGATGGAACATCCGGATGAACATCAGAACGTGCGTCAGCTGATTGGCAAACTGCATGAACGCCAACAAAATGTCCCGGTATTTCTGCTGGGCGATCGCGAAAAAGCGCTGGCGGCGATGGATCGCGACCTGCTAGAGCTGGTAGACGAATTTGCGTGGATTCTGGAAGATACCGCGGATTTTATTGCGGGCCGCGCAGTAGCTGCCATGACCCGCTATCGTCAGCAGCTGCTGCCGCCGCTGTTTAGCGCGCTGATGAAATATAGCGATATTCATGAATATAGCTGGGCGGCGCCGGGCCATCAAGGCGGCGTGGGCTTTACCAAAACCCCGGCGGGCCGCTTTTATCATGATTATTATGGCGAAAACCTGTTTCGCACCGATATGGGCATTGAACGCACGAGCCTGGGCAGCCTGCTGGATCATACCGGCGCGTTTGGCGAAAGCGAAAAATATGCGGCGCGCGTGTTTGGCGCGGATCGCAGCTGGAGCGTGGTGGTGGGCACGAGCGGCAGCAACCGCACCATTATGCAAGCGTGCATGACCGATAACGATGTGGTGGTGGTGGATCGCAACTGCCATAAAAGCATTGAACAAGGCCTGATGCTGACCGGCGCGAAACCGGTGTATATGGTGCCGAGCCGCAACCGCTATGGCATTATTGGCCCGATTTATCCGCAAGAAATGCAGCCGGAAACCCTGCAGAAGAAAATTAGCGAAAGCCCGCTGACCAAAGATAAAGCGGGTCAGAAACCGAGCTATTGCGTGGTGACCAACTGCACCTATGATGGCGTGTGCTATAACGCGAAAGAAGCGCAAGATCTGCTGGAAAAAACTAGCGATCGCCTGCATTTTGATGAAGCGTGGTATGGCTATGCGCGCTTTAACCCGATTTATGCGGATCATTATGCGATGCGCGGCGAACCGGGCGATCATAACGGCCCGACCGTGTTTGCGACCCATAGCACCCATAAACTGCTGAACGCGCTTTCGCAAGCGAGCTATATTCATGTGCGCGAAGGCCGCGGCGCGATTAACTTTAGCCGCTTTAACCAAGCGTATATGATGCATGCGACCACGAGCCCGCTGTATGCGATTTGCGCGAGCAACGATGTGGCGGTGAGCATGATGGATGGCAACAGCGGCCTGAGCCTGACCCAAGAAGTGATTGATGAAGCGGTGGATTTTCGCCAAGCGATGGCGCGCCTGTATAAAGAATTTACCGCGGATGGCAGCTGGTTTTTTAAACCGTGGAACAAAGAAGTGGTGACCGATCCGCAGACCGGCAAAACCTATGATTTTGCGCTGGCGCCGACCAAACTGCTGACCACCGTGCAAGATTGCTGGGTGATGCATCCGGGCGAAAGCTGGCATGGCTTTAAAGATATTCCGGATAACTGGAGCATGCTGGATCCGATTAAAGTGAGCATTCTGGCGCCGGGCATGGGCGAAGATGGAGAGCTGGAAGAAACGGGTGTGCCGGCGGCGCTGGTAACCGCGTGGTTGGGCCGCCATGGCATTGTGCCGACCCGCACCACCGATTTTCAGATTATGTTTCTGTTTAGCATGGGCGTGACCCGCGGCAAATGGGGCACCCTGGTGAACACCCTGTGCAGCTTTAAACGCCATTATGATGCGAACACCCCGCTGGCGCAAGTGATGCCGGAGCTGGTTGAGCAGTATCCGGATACCTATGCGAACATGGGCATTCATGATCTGGGCGATACCATGTTTGCGTGGCTGAAAGAAAACAACCCGGGCGCGCGCCTGAACGAAGCGTATAGCGGCCTGCCGGTGGCGGAAGTGACCCCGCGCGAAGCGTATAACGCGATTGTGGATAACAACGTGGAACTGGTGAGCATTGAAAACCTGCCGGGCCGCATTGCGGCGAACAGCGTGATTCCGTATCCGCCGGGCATTCCGATGCTGCTGAGCGGCGAAAACTTTGGCGATAAAAACAGCCCGCAAGTGAGCTATCTGCGCAGCCTGCAGAGCTGGGATCATCATTTTCCGGGCTTTGAACATGAAACCGAAGGCACCGAAATTATTGATGGCATTTATCATGTGATGTGCGTGAAAGCG |
| Linker | GGCGGCGGCGGCAGCGGCGGTGGCGGCAGC |
| YFP | ATGGTGAGCAAAGGCGAAGAACTGTTTACCGGCGTGGTGCCGATTCTGGTGGAACTGGATGGCGATGTGAACGGCCATAAATTTAGCGTGAGCGGCGAAGGCGAAGGCGATGCGACCTATGGCAAACTGACCCTGAAACTGATTTGCACCACCGGCAAACTGCCGGTGCCGTGGCCGACCCTGGTGACCACCTTTGGCTATGGCGTGCAGTGCTTTGCGCGCTATCCGGATCACATGCGTCAGCATGACTTTTTTAAAAGCGCGATGCCGGAAGGCTATGTGCAAGAACGCACCATCTTTTTTAAAGATGATGGCAACTATAAAACCCGCGCGGAAGTGAAATTTGAAGGCGATACCCTGGTGAACCGCATTGAACTGAAAGGCATTGATTTTAAAGAAGATGGCAACATTCTGGGCCATAAACTGGAATATAACTATAACAGCCATAACGTGTATATTATGGCGGATAAACAGAAAAACGGCATTAAAGTGAACTTTAAAATTCGCCATAACATTGAAGATGGCAGCGTGCAGCTGGCGGATCATTATCAGCAGAACACCCCGATTGGCGATGGCCCGGTGCTGCTGCCGGATAACCATTATCTGAGCTATCAGAGCGCGCTGAGCAAAGATCCGAACGAAAAACGCGATCACATGGTGCTGCTGGAATTTGTGACCGCGGCGGGCATTACCCTGGGCATGGATGAACTGTATAAA |
| mCherry | ATGGTGAGCAAGGGCGAGGAGGATAACATGGCCATCATCAAGGAGTTCATGCGCTTCAAGGTGCACATGGAGGGCTCCGTGAACGGCCACGAGTTCGAGATCGAGGGCGAGGGCGAGGGCCGCCCCTACGAGGGCACCCAGACCGCCAAGCTGAAGGTGACCAAGGGTGGCCCCCTGCCCTTCGCCTGGGACATCCTGTCCCCTCAGTTCATGTACGGCTCCAAGGCCTACGTGAAGCACCCCGCCGACATCCCCGACTACTTGAAGCTGTCCTTCCCCGAGGGCTTCAAGTGGGAGCGCGTGATGAACTTCGAGGACGGCGGCGTGGTGACCGTGACCCAGGACTCCTCCTTGCAGGACGGCGAGTTCATCTACAAGGTGAAGCTGCGCGGCACCAACTTCCCCTCCGACGGCCCCGTAATGCAGAAGAAGACCATGGGCTGGGAGGCCTCCTCCGAGCGGATGTACCCCGAGGACGGCGCCCTGAAGGGCGAGATCAAGCAGAGGCTGAAGCTGAAGGACGGCGGCCACTACGACGCTGAGGTCAAGACCACCTACAAGGCCAAGAAGCCCGTGCAGCTGCCCGGCGCCTACAACGTCAACATCAAGTTGGACATCACCTCCCACAACGAGGACTACACCATCGTGGAACAGTACGAACGCGCCGAGGGCCGCCACTCCACCGGCGGCATGGACGAGCTGTACAAG |
| sfGFP | ATGCGTAAAGGCGAAGAGCTGTTCACTGGTGTCGTCCCTATTCTGGTGGAACTGGATGGTGATGTCAACGGTCATAAGTTTTCCGTGCGTGGCGAGGGTGAAGGTGACGCAACTAATGGTAAACTGACGCTGAAGTTCATCTGTACTACTGGTAAACTGCCGGTACCTTGGCCGACTCTGGTAACGACGCTGACTTATGGTGTTCAGTGCTTTGCTCGTTATCCGGACCATATGAAGCAGCATGACTTCTTCAAGTCCGCCATGCCGGAAGGCTATGTGCAGGAACGCACGATTTCCTTTAAGGATGACGGCACGTACAAAACGCGTGCGGAAGTGAAATTTGAAGGCGATACCCTGGTAAACCGCATTGAGCTGAAAGGCATTGACTTTAAAGAAGACGGCAATATCCTGGGCCATAAGCTGGAATACAATTTTAACAGCCACAATGTTTACATCACCGCCGATAAACAAAAAAATGGCATTAAAGCGAATTTTAAAATTCGCCACAACGTGGAGGATGGCAGCGTGCAGCTGGCTGATCACTACCAGCAAAACACTCCAATCGGTGATGGTCCTGTTCTGCTGCCAGACAATCACTATCTGAGCACGCAAAGCGTTCTGTCTAAAGATCCGAACGAGAAACGCGATCATATGGTTCTGCTGGAGTTCGTAACCGCAGCGGGCATCACGCATGGTATGGATGAACTGTACAAA |
| TFX | ATGGCCGTCACATCCAACGAGACCGGGTACCACGACGGGTACTTCTACTCGTTCTGGACCGTCGCGCCCGGAACGGTCTCCATGGAGCTGGGCCCTGGCGGAAACTACAGCACCTCCTGGCGGAACACCGGGAACTTCGTCGCCGGTAAGGGATGGGCCACCGGTGGCCGCCGGACCGTGACCTACTCCGCCTGCTTCAACCCGTCGGGTAACGCCTACCTGACCCTCTACGGGTGGACGCGGAACCCGCTCGTGGAGTACTACATCGTCGAAAGCTGGGGCACCTACCGGCCCACCGGTACCTACATGGGCACGGTGACCACCGACGGTGGTACCTACGACATCTACAAGACCACGCGGTACAACGCGCCCTCCATCGAAGGCACCCGGACCTTCGACCAGTACTGGAGCGTCCGCCAGTCCAAGCGGACCAGCGGTACCATCACCGCGGGGAACCACTTCGACGCGTGGGCCCGCCACGGTATGCACCTCGGAACCCACGACTACATGATCATGGCGACCGAGGGCTACCAGAGCAGCGGATCCTCCAACGTGACGCTGGGCACCAGCGGCGGTGACAAC |

**Table S2** The amino acid sequences used in this study.

| Name | Amino acid sequence |
| --- | --- |
| LdcI | MNVIAILNHMGVYFKEEPIRELHRALERLNFQIVYPNDRDDLLKLIENNARLCGVIFDWDKYNLELCEEISKMNENLPLYAFANTYSTLDVSLNDLRLQISFFEYALGAAEDIANKIKQTTDEYINTILPPLTKALFKYVREGKYTFCTPGHMGGTAFQKSPVGSLFYDFFGPNTMKSDISISVSELGSLLDHSGPHKEAEQYIARVFNADRSYMVTNGTSTANKIVGMYSAPAGSTILIDRNCHKSLTHLMMMSDVTPIYFRPTRNAYGILGGIPQSEFQHATIAKRVKETPNATWPVHAVITNSTYDGLLYNTDFIKKTLDVKSIHFDSAWVPYTNFSPIYEGKCGMSGGRVEGKVIYETQSTHKLLAAFSQASMIHVKGDVNEETFNEAYMMHTTTSPHYGIVASTETAAAMMKGNAGKRLINGSIERAIKFRKEIKRLRTESDGWFFDVWQPDHILTTECWPLRSDSTWHGFKNIDNEHMYLDPIKVTLLTPGMEKDGTMSDFGIPASIVAKYLDEHGIVVEKTGPYNLLFLFSIGIDKTKALSLLRALTDFKRAFDLNLRVKNMLPSLYREDPEFYENMRIQELAQNIHKLIVHHNLPDLMYRAFEVLPTMVMTPYAAFQKELHGMTEEVYLDEMVGRINANMILPYPPGVPLVMPGEMITEESRPVLEFLQMLCEIGAHYPGFETDIHGAYRQADGRYTVKVLKEESKK |
| AsnC | MENYLIDNLDRGILEALMGNARTAYAELAKQFGVSPETIHVRVEKMKQAGIITGARIDVSPKQLGYDVGCFIGIILKSAKDYPSALAKLESLDEVTEAYYTTGHYSIFIKVMCRSIDALQHVLINYIQTIYEIQSTETLIVLQNPIMRTIKP |
| IadA | MIDYTAAGFTLLQGAHLYAPEDRGICDVLVANGKIIAVASNIPSDIVPNCTVVDLSGQILCPGFIDQHVHLIGGGGEAGPTTRTPEVALSRLTEAGVTSVVGLLGTDSISRHPESLLAKTRALNEEGISAWMLTGAYHVPSRTITGSVEKDVAIIDRVIGVKCAISDHRSAAPDVYHLANMAAESRVGGLLGGKPGVTVFHMGDSKKALQPIYDLLENCDVPISKLLPTHVNRNVPLFYQALEFARKGGTIDITSSIDEPVAPAEGIARAVQAGIPLARVTLSSDGNGSQPFFDDEGNLTHIGVAGFETLLETVQVLVKDYDFSISDALRPLTSSVAGFLNLTGKGEILPGNDADLLVMTPELRIEQVYARGKLMVKDGKACVKGTFETA |
| FucU | MLKTISPLISPELLKVLAEMGHGDEIIFSDAHFPAHSMGPQVIRADGLLVSDLLQAIIPLFELDSYAPPLVMMAAVYGDTLDPEVERRYRNALSLQAPCPDIIRINRFAFYERAQKAFAIVITGERAKYGNILLKKGVTP |
| PanB | MKPTTISLLQKYKQEKKRFATITAYDYSFAKLFADEGLNVMLVGDSLGMTVQGHDSTLPVTVADIAYHTAAVRRGAPNCLLLADLPFMAYATPEQAFENAATVMRAGANMVKIEGGEWLVETVQMLTERAVPVCGHLGLTPQSVNIFGGYKVQGRGLLAGLQLLSDALALEAAGAQLLVLECVPVELAKRITEALAIPVIGIGAGNVTDGQILVMHDAFGITGGHIPKFAKNFLAETGDIRAAVRQYMAEVESGVYPGEEHSF |
| AdiA | MKVLIVESEFLHQDTWVGNAVERLADALSQQNVTVIKSTSFDDGFAILSSNEAIDCLMFSYQMEHPDEHQNVRQLIGKLHERQQNVPVFLLGDREKALAAMDRDLLELVDEFAWILEDTADFIAGRAVAAMTRYRQQLLPPLFSALMKYSDIHEYSWAAPGHQGGVGFTKTPAGRFYHDYYGENLFRTDMGIERTSLGSLLDHTGAFGESEKYAARVFGADRSWSVVVGTSGSNRTIMQACMTDNDVVVVDRNCHKSIEQGLMLTGAKPVYMVPSRNRYGIIGPIYPQEMQPETLQKKISESPLTKDKAGQKPSYCVVTNCTYDGVCYNAKEAQDLLEKTSDRLHFDEAWYGYARFNPIYADHYAMRGEPGDHNGPTVFATHSTHKLLNALSQASYIHVREGRGAINFSRFNQAYMMHATTSPLYAICASNDVAVSMMDGNSGLSLTQEVIDEAVDFRQAMARLYKEFTADGSWFFKPWNKEVVTDPQTGKTYDFALAPTKLLTTVQDCWVMHPGESWHGFKDIPDNWSMLDPIKVSILAPGMGEDGELEETGVPAALVTAWLGRHGIVPTRTTDFQIMFLFSMGVTRGKWGTLVNTLCSFKRHYDANTPLAQVMPELVEQYPDTYANMGIHDLGDTMFAWLKENNPGARLNEAYSGLPVAEVTPREAYNAIVDNNVELVSIENLPGRIAANSVIPYPPGIPMLLSGENFGDKNSPQVSYLRSLQSWDHHFPGFEHETEGTEIIDGIYHVMCVKA |
| YFP | MVSKGEELFTGVVPILVELDGDVNGHKFSVSGEGEGDATYGKLTLKLICTTGKLPVPWPTLVTTFGYGVQCFARYPDHMRQHDFFKSAMPEGYVQERTIFFKDDGNYKTRAEVKFEGDTLVNRIELKGIDFKEDGNILGHKLEYNYNSHNVYIMADKQKNGIKVNFKIRHNIEDGSVQLADHYQQNTPIGDGPVLLPDNHYLSYQSALSKDPNEKRDHMVLLEFVTAAGITLGMDELYK |
| sfGFP | MRKGEELFTGVVPILVELDGDVNGHKFSVRGEGEGDATNGKLTLKFICTTGKLPVPWPTLVTTLTYGVQCFARYPDHMKQHDFFKSAMPEGYVQERTISFKDDGTYKTRAEVKFEGDTLVNRIELKGIDFKEDGNILGHKLEYNFNSHNVYITADKQKNGIKANFKIRHNVEDGSVQLADHYQQNTPIGDGPVLLPDNHYLSTQSVLSKDPNEKRDHMVLLEFVTAAGITHGMDELYK |
| mCherry | MVSKGEEDNMAIIKEFMRFKVHMEGSVNGHEFEIEGEGEGRPYEGTQTAKLKVTKGGPLPFAWDILSPQFMYGSKAYVKHPADIPDYLKLSFPEGFKWERVMNFEDGGVVTVTQDSSLQDGEFIYKVKLRGTNFPSDGPVMQKKTMGWEASSERMYPEDGALKGEIKQRLKLKDGGHYDAEVKTTYKAKKPVQLPGAYNVNIKLDITSHNEDYTIVEQYERAEGRHSTGGMDELYK |
| TFX | MAVTSNETGYHDGYFYSFWTVAPGTVSMELGPGGNYSTSWRNTGNFVAGKGWATGGRRTVTYSACFNPSGNAYLTLYGWTRNPLVEYYIVESWGTYRPTGTYMGTVTTDGGTYDIYKTTRYNAPSIEGTRTFDQYWSVRQSKRTSGTITAGNHFDAWARHGMHLGTHDYMIMATEGYQSSGSSNVTLGTSGGDN |

**Table S3** Fluorescence ratios of six proteins fused with YFP at the N-terminus to YFP at the C-terminus.

| protein | LdcI | AsnC | IadA | FucU | PanB | AdiA |
| --- | --- | --- | --- | --- | --- | --- |
| Total protein fluorescence ratio | 3.19 | 6.84 | 4.24 | 3.33 | 1.53 | 2.38 |
| Soluble protein fluorescence ratio | 12.80 | 14.51 | 34.32 | 62.76 | 26.27 | 5.86 |
| Solubility ratio | 4.02 | 2.12 | 8.09 | 18.87 | 17.22 | 2.46 |

**Table S4** Structural information of six proteins.

| Protein name | PDB ID | Number of subunits | Symmetry | Uniprot ID |
| --- | --- | --- | --- | --- |
| Lysine decarboxylase (LdcI) | 3N75 | 10 | D5 | P0A9H3 |
| Regulatory protein AsnC (AsnC) | 2CG4 | 8 | D4 | P0ACI6 |
| Isoaspartyl dipeptidase (IadA) | 1POK | 8 | D4 | P39377 |
| L-fucose mutarotase (FucU) | 2WCV | 10 | D5 | P0AEN8 |
| 3-methyl-2-oxobutanoate hydroxymethyltransferase (PanB) | 1M3U | 10 | D5 | P31057 |
| Biodegradative arginine decarboxylase (AdiA) | 2VYC | 10 | D5 | P28629 |


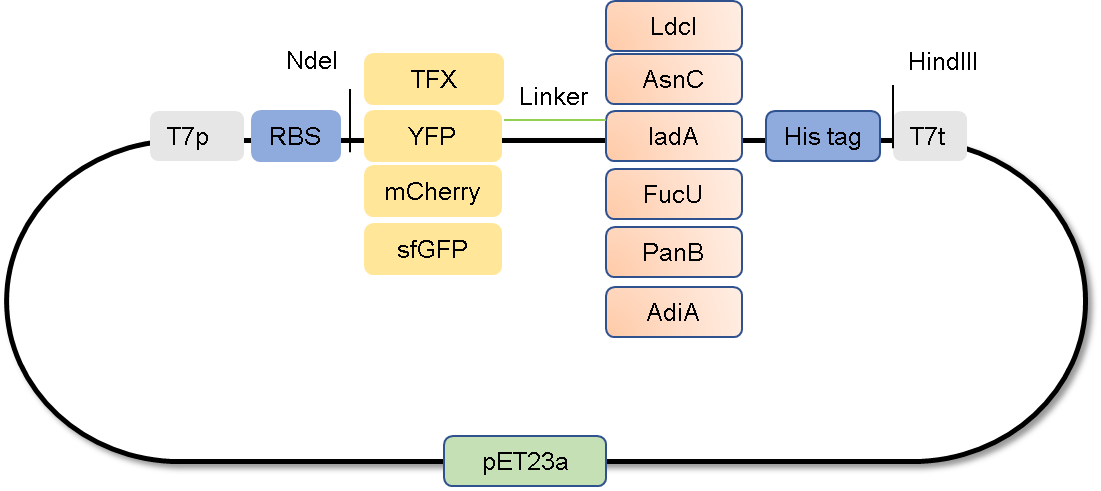


**Fig. S1** The protein construct design diagram.

**Fig. S2** The plasmid map of pET23a-mCherry-FucU. The mCherry-FucU sequence was located between RBS and T7 terminator on the pET-23a.

**Fig. S3** The plasmid map of pET23a-sfGFP-FucU. The sfGFP-FucU sequence was located between RBS and T7 terminator on the pET-23a.

**Fig. S4** The plasmid map of pET23a-TFX-FucU. The TFX-FucU sequence was located between RBS and T7 terminator on the pET-23a.

**Fig. S5** The plasmid map of pET23a-YFP-FucU. The YFP-FucU sequence was located between RBS and T7 terminator on the pET-23a.

**Fig. S6** The plasmid map of pET23a-FucU-YFP. The FucU-YFP sequence was located between RBS and T7 terminator on the pET-23a.

**
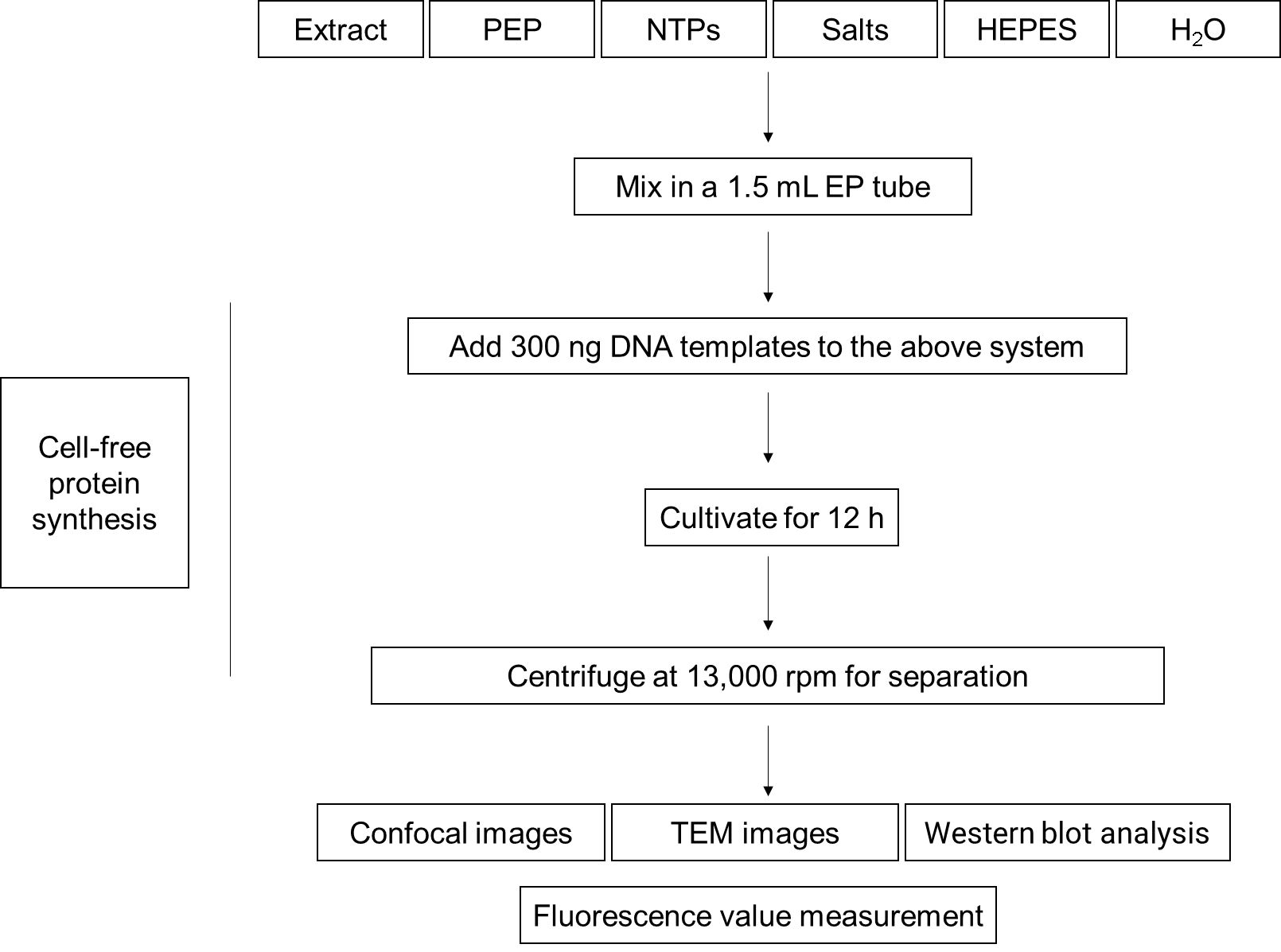
Fig. S7** The workflow of cell-free protein synthesis system. This workflow was used to study the effects of fusion proteins and to investigate the solubility of these proteins.


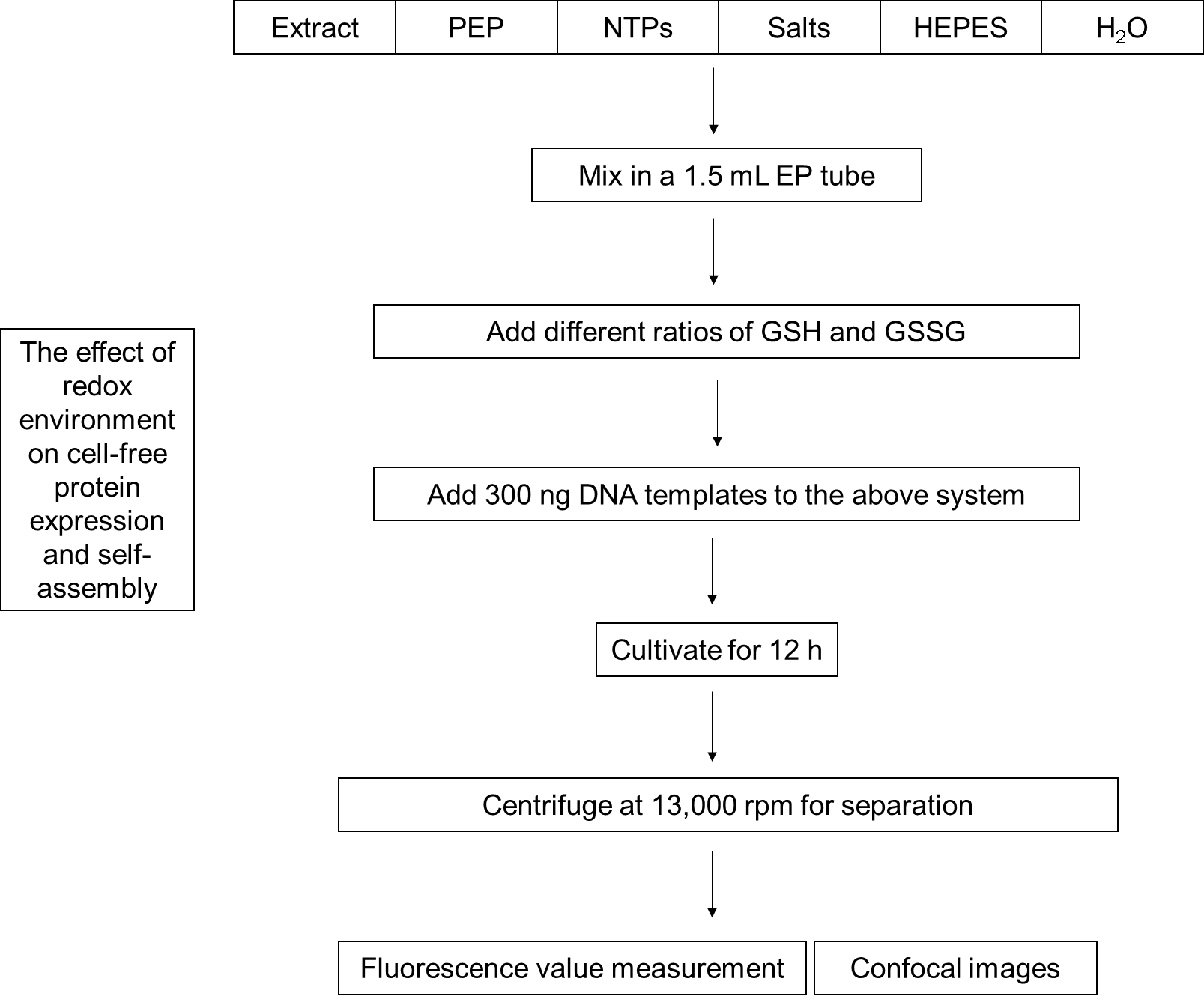


**Fig. S8** The workflow for the effects of redox environments on cell-free protein expression and self-assembly. This workflow was used to investigate the effects of redox conditions on the synthesis and self-assembly of six proteins.


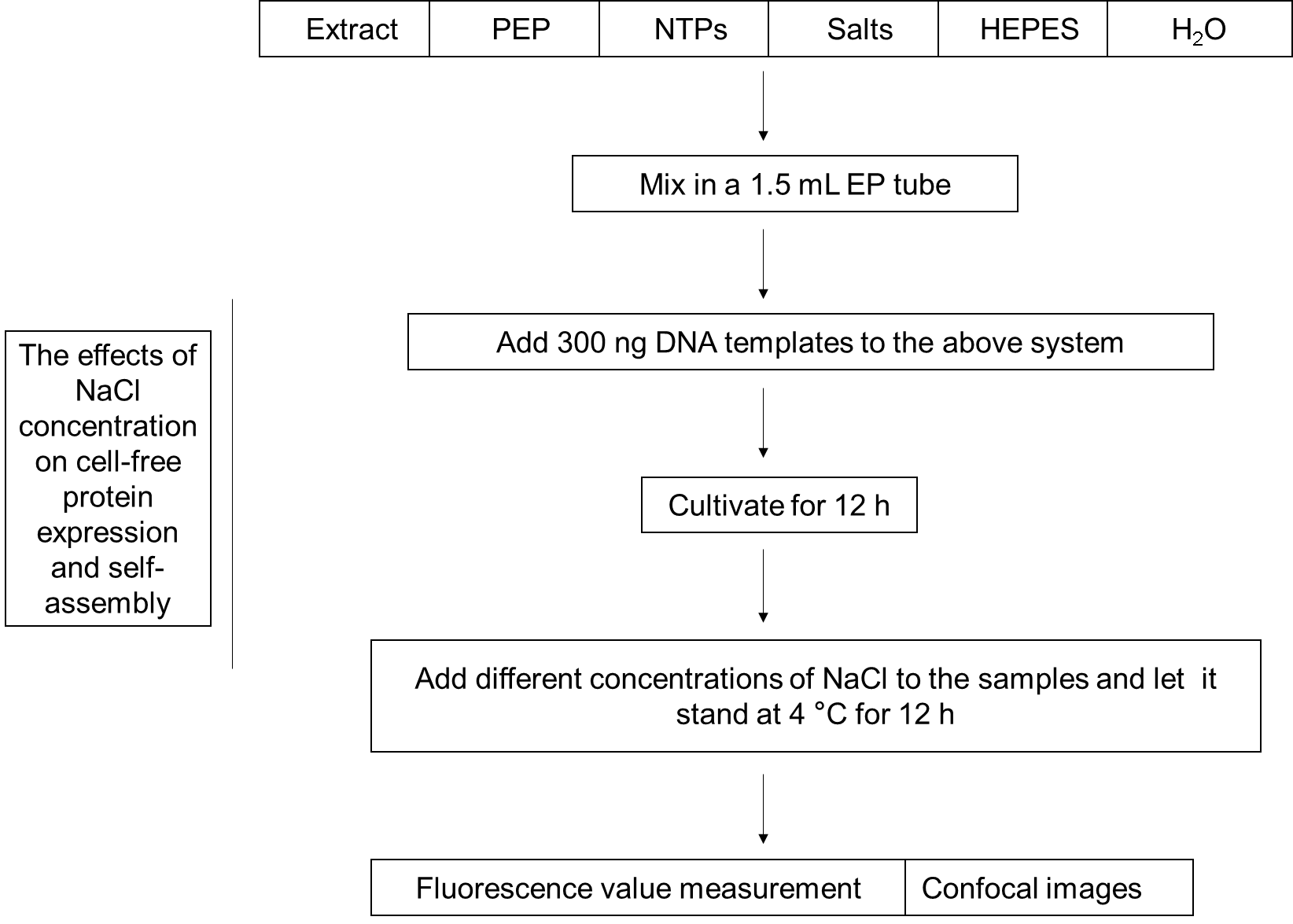


**Fig. S9** The workflow for the effects of NaCl concentration on cell-free protein expression and self-assembly. This workflow was used to investigate the effect s of NaCl concentration on the synthesis and self-assembly of six proteins.


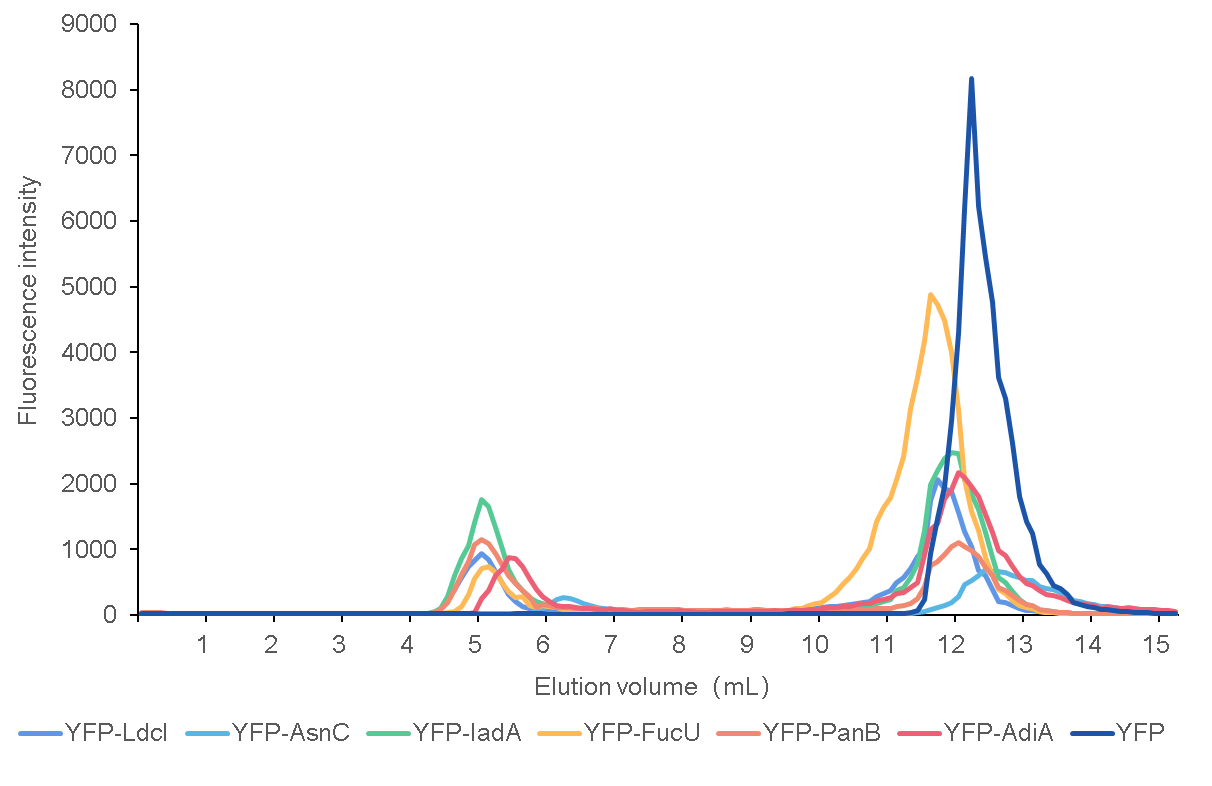


**Fig. S10** The size-exclusion chromatography results of six protein constructs produced in the CFPS systems. All six proteins formed polymers with larger sizes than monomer YFP protein.


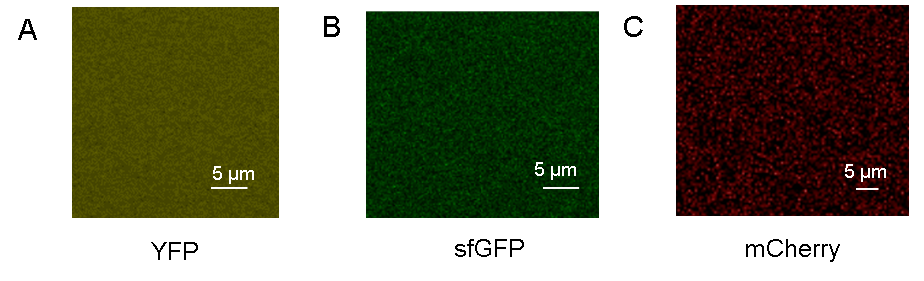


**Fig. S11** Imaging of YFP, sfGFP, mCherry proteins expressed individually in CFPS system under confocal microscope.


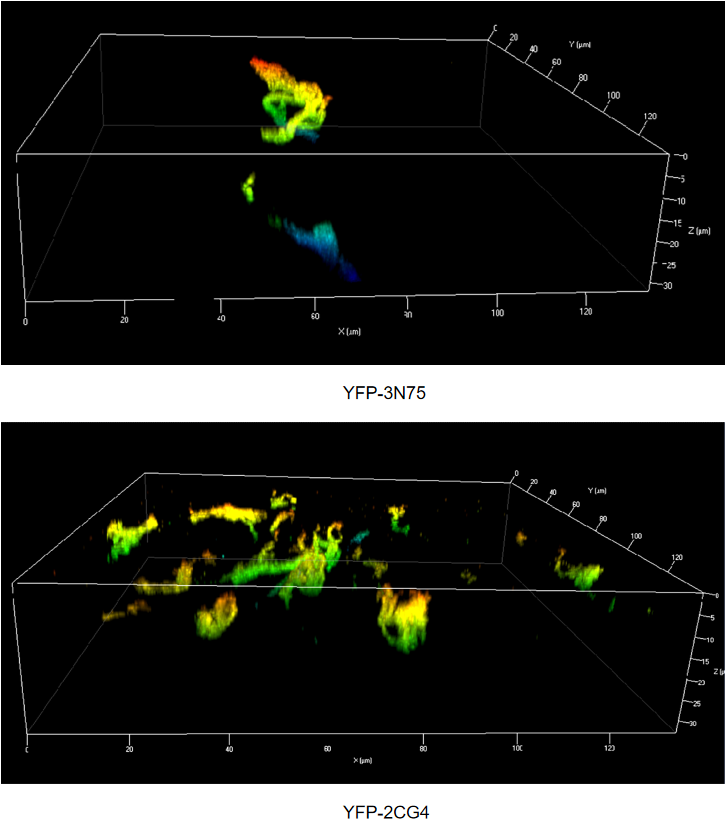


**Fig. S12** Imaging of the self-assembled protein YFP-LdcI under a confocal microscope. This protein was synthesized and assembled *in situ* in the CFPS system.


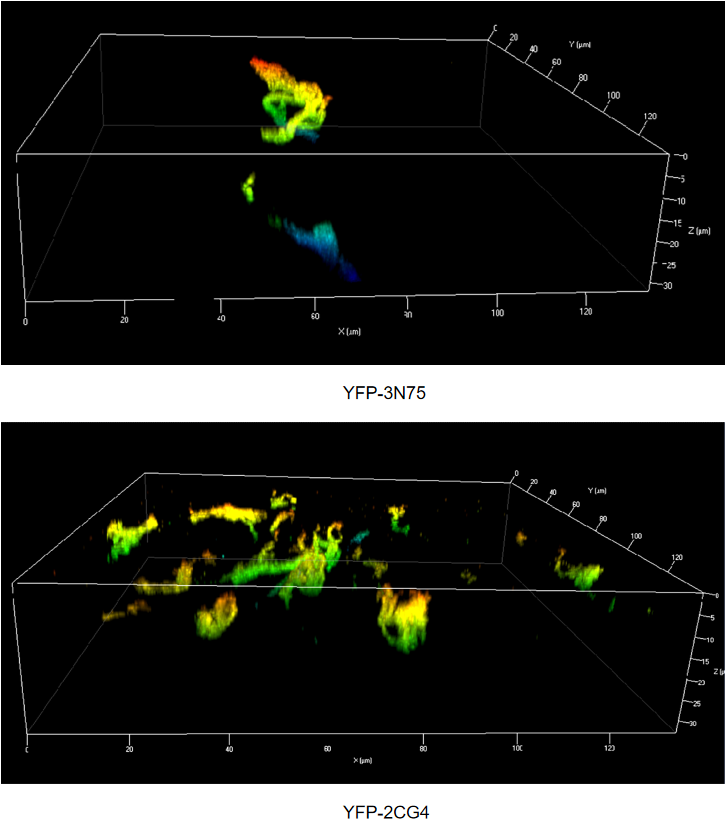


**Fig. S13** Imaging of the self-assembled protein YFP-AsnC under a confocal microscope. This protein was synthesized and assembled *in situ* in the CFPS system.


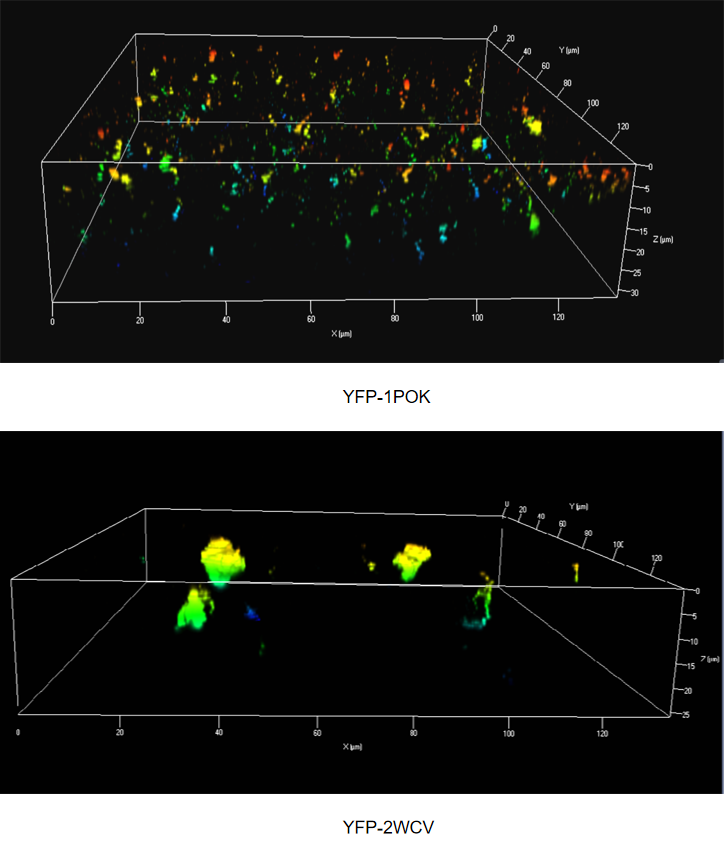


**Fig. S14** Imaging of the self-assembled protein YFP-IadA under a confocal microscope. This protein was synthesized and assembled *in situ* in the CFPS system.


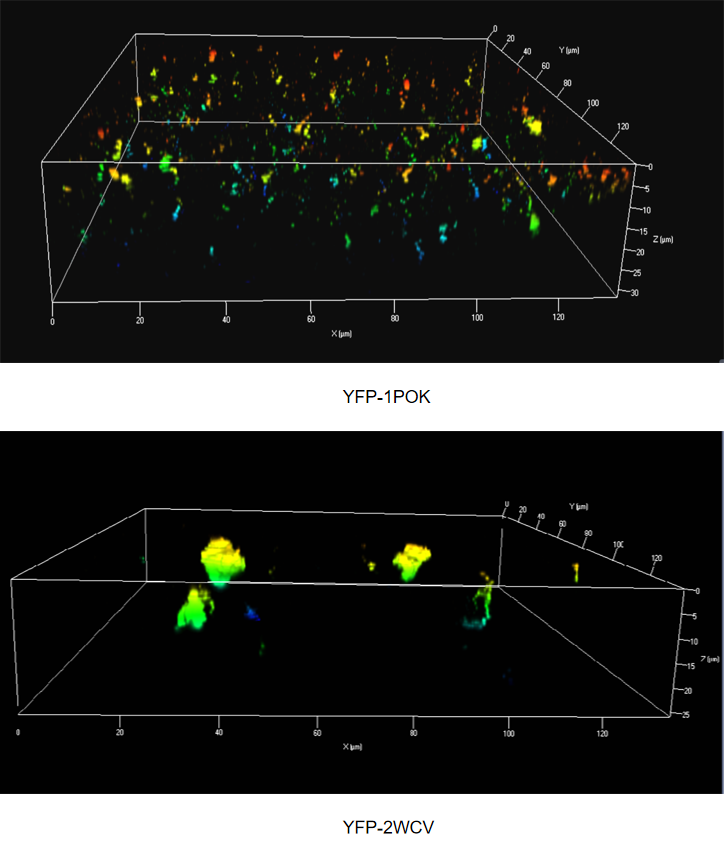


**Fig. S15** Imaging of the self-assembled protein YFP-FucU under a confocal microscope. This protein was synthesized and assembled *in situ* in the CFPS system.


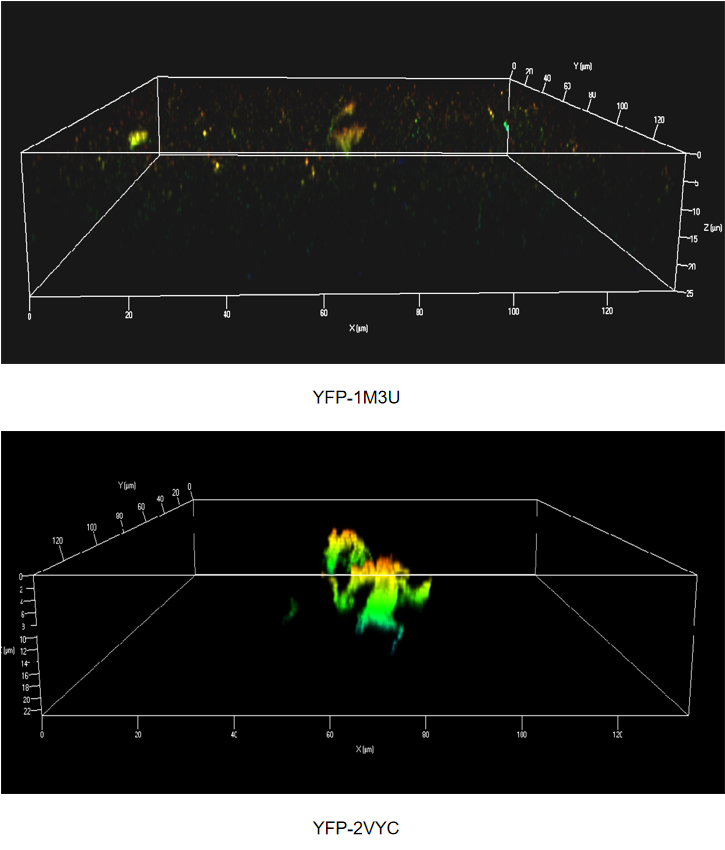


**Fig. S16** Imaging of the self-assembled protein YFP-PanB under a confocal microscope. This protein was synthesized and assembled *in situ* in the CFPS system.


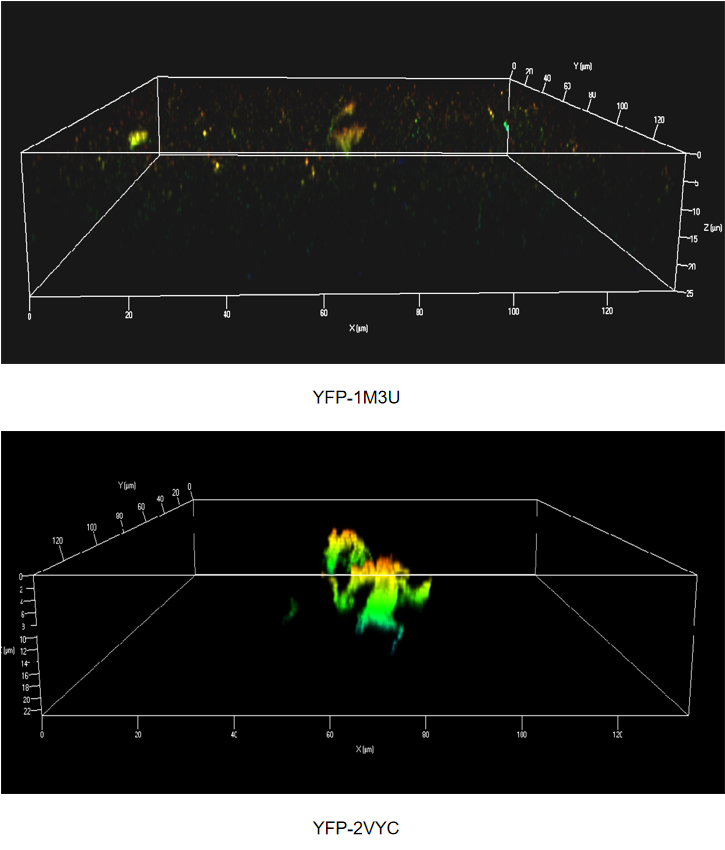


**Fig. S17** Imaging of the self-assembled protein YFP-AdiA under a confo1POK2WCVcal microscope. This protein was synthesized and assembled *in situ* in the CFPS system.


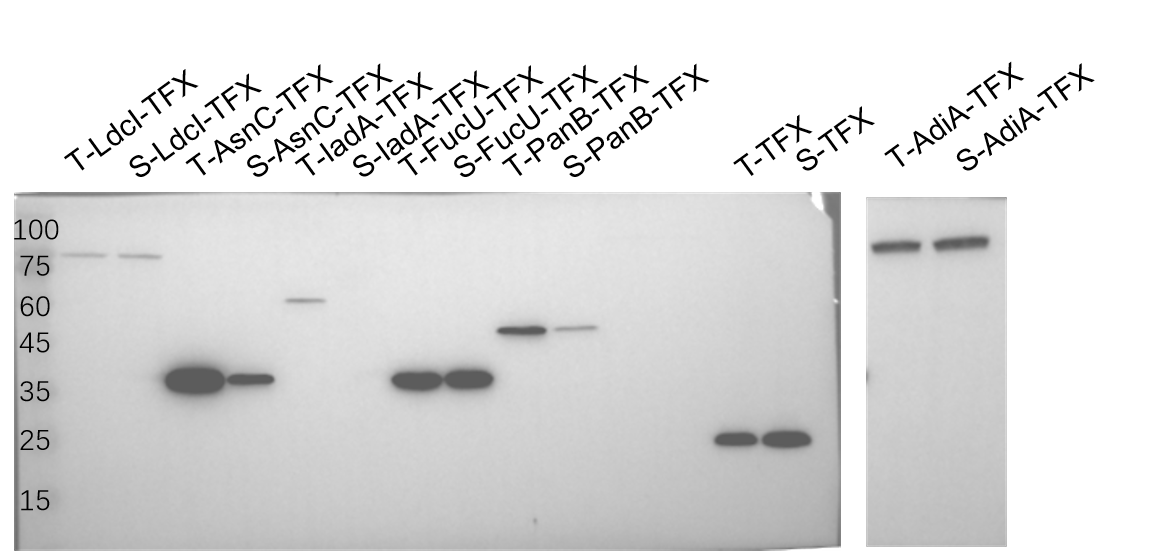


**Fig. S18** Western blot analysis of proteins fused with TFX. These proteins were synthesized and assembled *in situ* in the CFPS system.
